# Exploration–Exploitation Tradeoff in the Adaptive Information Sampling of Unknown Spatial Fields with Mobile Robots

**DOI:** 10.3390/s23239600

**Published:** 2023-12-04

**Authors:** Aiman Munir, Ramviyas Parasuraman

**Affiliations:** School of Computing, University of Georgia, Athens, GA 30602, USA; aiman.munir@uga.edu

**Keywords:** mobile robots, exploration, informative path planning, adaptive sampling, mapping

## Abstract

Adaptive information-sampling approaches enable efficient selection of mobile robots’ waypoints through which the accurate sensing and mapping of a physical process, such as the radiation or field intensity, can be obtained. A key parameter in the informative sampling objective function could be optimized balance the need to explore new information where the uncertainty is very high and to exploit the data sampled so far, with which a great deal of the underlying spatial fields can be obtained, such as the source locations or modalities of the physical process. However, works in the literature have either assumed the robot’s energy is unconstrained or used a homogeneous availability of energy capacity among different robots. Therefore, this paper analyzes the impact of the adaptive information-sampling algorithm’s information function used in exploration and exploitation to achieve a tradeoff between balancing the mapping, localization, and energy efficiency objectives. We use Gaussian process regression (GPR) to predict and estimate confidence bounds, thereby determining each point’s informativeness. Through extensive experimental data, we provide a deeper and holistic perspective on the effect of information function parameters on the prediction map’s accuracy (RMSE), confidence bound (variance), energy consumption (distance), and time spent (sample count) in both single- and multi-robot scenarios. The results provide meaningful insights into choosing the appropriate energy-aware information function parameters based on sensing objectives (e.g., source localization or mapping). Based on our analysis, we can conclude that it would be detrimental to give importance only to the uncertainty of the information function (which would explode the energy needs) or to the predictive mean of the information (which would jeopardize the mapping accuracy). By assigning more importance to the information uncertainly with some non-zero importance to the information value (e.g., 75:25 ratio), it is possible to achieve an optimal tradeoff between exploration and exploitation objectives while keeping the energy requirements manageable.

## 1. Introduction

The mobile robot-aided mapping of environmental processes, such as information sampling [1], sensor coverage [2], localization of source [3], and monitoring of environmental phenomena [4], has been well investigated. In particular, sensor coverage with multiple robots involves optimally positioning robots to maximize overall performance in terms of sensing environmental phenomena. Monitoring is a persistent process for identifying anomalies in physical processes by efficiently collecting the most informative samples. For all of these objectives, it is required to obtain the model of the underlying processes through the physical sampling or mapping of the environment.

Modeling of physical processes plays an important role in autonomous robots’ decision-making. Robots must create a model of the environmental phenomenon to accomplish mapping tasks, especially when the environment is unexplored [5]. Mapping spatial distribution enables the robots to work autonomously in search and rescue missions and make decisions without human intervention (e.g., for rescuing targets in areas with high radiation exposure). Similarly, the robot requires knowing the areas with higher risks to always choose a path to connect to the network, maintain communication, etc. [6,7]. Therefore, robotics researchers are actively investigating different strategies for mapping physical processes, such as radiation, Wi-Fi signal strength, gas distribution, radio signal strength, etc., using unmanned vehicles [3,8,9].

To map the environmental phenomenon, sensing and measuring the value of a physical process throughout the environment is crucial. However, not all the locations can provide helpful information about the change in the process itself. The primary considerations involved in mapping such processes include the degree of autonomy, accuracy, and efficiency. Measuring intensity at every location is deemed impractical; hence, dense sampling is not viable in mapping. Instead, an accurate, time- and cost-effective process model can be obtained by gathering samples from the points containing the most significant information.

Exploration refers to accumulating samples from previously unexplored areas to reduce uncertainty in the map, while exploitation implies determining the next sampling point based on the best information from the current estimates (to localize the source, for example). Mapping algorithms and techniques in the literature use either exploration or exploitation, or a combination of both. For example, an active control law for mobile robots is proposed in [3] to shift between exploration and exploitation objectives; research in [2] has utilized a utility function to adjust exploration and exploitation. On the other hand, a parallel strip route (pure exploratory approach) is used in [10,11] to explore the environment for mapping the spatial distribution. Hence, these two techniques (exploration and exploitation) are fundamental to the mapping process. The study in this paper aims to compare how well various exploration and exploitation techniques perform, as well as analyze how tradeoffs between exploring and exploitation affect different sampling objectives. Specifically, the contributions made in this paper are three-fold.

- We comprehensively compare various information-sampling variants. Our analysis evaluates the balance of performance metrics related to the accuracy, confidence bound, time, and energy consumption for the exploration of objective and source localization accuracy for the exploitation objective. Additionally, we investigate how both objectives can be balanced.
- We systematically analyze the impact of different source locations on this tradeoff using single-robot experiments with random walk (RW) and fixed sweep trajectory (FS) as the baselines for comparison.
- We extend this analysis to multi-robot settings with fixed and dynamic Voronoi partition-based adaptive sampling [12] assignments to each robot in the system.

The outcomes of this investigation provide significant perspectives on selecting appropriate weights in the information function for active sampling with mobile robots, especially in scenarios where it is necessary to strike a balance between exploration (ensuring well-balanced performance metrics) and exploitation (locating sources with minimal samples) objectives.

## 2. Related Work

In informative path planning, both adaptive (taking the informativeness of the sampled data into account) and non-adaptive (without considering informativeness) sampling approaches have been previously reported in the literature. Non-adaptive sampling methods focus on sampling the whole environment [11,13,14]. Non-adaptive methods are time-consuming, and with such methods, it is hard to achieve the desired threshold (upper bound) of information certainty. Alternatively, adaptive sampling methods provide convergence to an objective (threshold), and sampling can be stopped as soon as the desired threshold is reached.

Table 1 provides detailed information about closely related works in the literature on adaptive information gathering. Several objective functions have been used in coordination with Gaussian process regression [15] to map the physical process (i.e., to predict the samples at unvisited (unexplored) locations with confidence bounds).

The approaches to planning a robot path that contains the most informative samples have been well studied. The technique utilized in [1] aimed to maximize the mean entropy information metric when searching for a station. An informative planner algorithm based on RRT was utilized to select the path that provides maximum utility, i.e., the tradeoff between the informativeness and cost to reach that station. However, the tradeoff was applied based on the budget; if the cost was lower than the budget, then the path was selected. The authors of [4] employed entropy as an information criterion over the Sparse Gaussian Process to identify the most informative locations to persistently monitor salinity in the ocean. Similarly, Ref. [19] used wireless signals for robot localization in an indoor GPS-denied environment. A path loss model was learned from the data, and then the Gaussian process was trained with the mismatches between the models and the data with a focus on better prediction of model variance. In [8], the authors focused on mapping in structured environments. The algorithm partitioned the environment for each robot and used differential entropy as an information theory metric on top of Gaussian Process predictions to determine the next sampling point.

The authors in [20] proposed a Hexagonal Tree (HexTree)-based sampling algorithm, which took samples over a set of hexagonal grid points and built a tree of possible trajectories by extending candidate trajectories toward the sampled points. In [24], an energy-aware approach is introduced to balance between coverage and sampling. Similarly, a recent work in [25] considered balancing the coverage and sampling (learning environmental model) objectives with a time-varying parameter. However, we consider adaptive sampling as an independent objective without the need for performing the area coverage of the environment, allowing us to focus solely on analyzing the informative sampling tradeoff with different objectives (exploration to obtain new data or exploitation of available data to complete the sampling objective).

Researchers have investigated decentralized methods for modeling the environmental process as well [26,27,28,29]. Ref. [26] introduced a technique utilizing radial basis functions, emphasizing the cooperative learning of the model under communication constraints. Meanwhile, Ref. [27] devised a multi-robot algorithm that employed a pure exploration strategy to map the underlying physical process using Spatial GPR. However, for our analysis, we chose a centralized server to mitigate potential biases associated with decentralization.

In a related study [28], a decentralized informative path-planning algorithm was introduced, aiming to balance exploration and exploitation. The study also conducted a comparison of exploitation coefficients based on completion time and mapping accuracy. However, the efficiency of the algorithm is influenced by the robot’s starting position, a factor not thoroughly examined, particularly in a multi-robot setting with a constrained energy perspective. Our study differs from [28] in that we holistically consider the energy consumption and confidence bound into account for the tradeoff analysis of different sampling objectives. We also report the effect of the information sampling parameter (coefficient) at different time instances during the exploration task. To avoid bias from the robot’s location or the source, we performed extensive analysis through simulation experiments with five different source locations and performed five trials per source location.

Jang et al. [29] proposed an approach to learn the underlying model function employing decentralized GPR. The paper implemented a pure exploration strategy to decrease variance, shifting to pure exploitation when the model’s variance exceeded a specified threshold. However, a potential drawback of this technique is that exploitation will not be initiated until the variance threshold is reached. In practical settings, determining the accurate threshold value is challenging without specific knowledge of the environmental process.

In the context of a time-invariant physical process, exploitation proves more advantageous than exploration, offering valuable insight, such as identifying the location of the source and determining its intensity. Our study aims to comprehensively understand which values for the exploration and exploitation coefficients yield more favorable tradeoffs for their respective underlying objectives without relying on a predefined threshold.

Compared to the literature, our work is novel and unique in the following ways: (1) we extensively analyze several variants of adaptive sampling parameters through simulation experiments with multiple source locations and distributions; (2) we present the analysis from a pure sensing perspective (i.e., to obtain an accurate prediction of the sensed information throughout the environment) with both single-robot and multi-robot use cases; and (3) we discuss how to holistically balance between sampling and energy demands considering practical resource constraints.

## 3. Robot Sampling Aided by Gaussian Processes Regression

Gaussian process regression (GPR) has been widely utilized for modeling spatial processes. For example, Ref. [30] used GPR to model spatial functions for mobile wireless sensor networks and to generate a likelihood model for signal strength measurements. To obtain a spatial map in an environment with limited communication, Ref. [12] employed GPR to the occurrence of model algae bloom. GPR was utilized in [3] to obtain a model of radio signal strength and to obtain the maximum likelihood of the source location. Therefore, we use GPR in our work to dynamically predict and continuously update the sensor data estimates for the whole map region (including unexplored locations) using the available data sampled so far from locations previously visited by the mobile robot.

A Gaussian process (GP) is a non-parametric continuous function that defines a probability distribution over functions. It assumes that every point has a normal distribution and that there is a correlation between values at these points. Let *q* be the 2D location (x and y coordinates) where $q\in Q\subset R^{2}$ from where the signal strength is measured, and let *z* be the measurement. The value of *z* at any location q can be related to a function f(q) using the Gaussian noise model for the observed location *q* as

(1) $$z=f\left(q\right)+\varepsilon ,$$

where ε is additive Gaussian noise.

We are interested in calculating a posterior function $f_{★}$ that makes predictions for given test locations $q_{★}\in Q_{★}\subset R^{2}$. A GPR model, also known as Kriging, assumes a GP prior that can be completely defined using mean and covariance. The joint Gaussian distribution on the test set $Q_{*}$, assuming noisy observation *z*, can be defined as follows:

(2) $$\left[\begin{array}{c} z\\ f_{*} \end{array}\right]∼N\left(\mu \left(q\right),\left[\begin{array}{cc} K+\sigma_{n}^{2}I & k_{*}\\ k_{*}^{T} & k_{**} \end{array}\right]\right),$$

where *K* is the covariance matrix between the training points, $k_{*}$ is the covariance matrix between the training points and test points, and $k_{**}$ is the covariance between only the test points. The posterior mean and variance for any testing location $q_{★}$ learned by GPR are as follows:

(3) $$\mu \left[f_{★}\right]=m\left(q\right)+k_{★}^{T}{\left(K+\sigma_{n}^{2}I\right)}^{-1}\left(y-m\left(q\right)\right)$$

(4) $$\sigma^{2}\left[f_{★}\right]=k_{★★}-k_{★}^{T}{\left(K+\sigma_{n}^{2}I\right)}^{-1}k_{★}$$

In our experiments, we employ a widely used kernel, i.e., a squared exponential.

(5) $$k\left(q,q^{\prime }\right)=\sigma_{f}^{2}\exp\left(-\frac{1}{2l^{2}}{\left|q-q^{\prime }\right|}^{2}\right)$$

where *q* and $q^{\prime }$ are both training points and $\sigma_{f}$ and l are hyper-parameters, called variance and length, respectively. We continuously learn these hyperparameters by maximizing the log marginal likelihood of the observations [3,31]. The variance and mean in Equations (3) and (4) are used to calculate the informativeness of every point.

The data used for training correspond to the information collected by the robot up to this point, while the testing data in this specific scenario encompass all the locations within the environment that the robot has not yet visited but needs to predict the signals for so it can decide which location it can go to next to speed up the sampling process. The methodology utilizes the fact that, once GPR is trained, mean values can be extrapolated for the entire region of interest.

### Information Sampling Using GPR

A method for determining the next sampling point using a utility function during the model-creation process is termed adaptive information sampling [8,32]. Depending on the criteria for selecting the next sampling location, adaptive sampling can be exploration-based, exploitation-based, or a mixture of both types. In this paper, we have used two non-adaptive sampling approaches that do not use an information function as baselines: a predefined S-shaped sweep trajectory (ordered zigzag pattern) (FS) and a random walk (RW). We have combined a random walk baseline with different variants of the adaptive information sampling to get an idea of the environmental process around the robot and then choose the next waypoint based on the information. Here, the first few samples are obtained out using RW, and then the adaptive sample follows. We use the Gaussian Process Upper Confidence Bound (GP-UCB) [33] information (utility) function to calculate the informativeness at every point *q*:

(6) $$I\left(q\right)=\alpha \mu_{q}+\beta \sigma_{q}^{2}.$$

Here, I(q), $\mu_{q}$ and $\sigma_{q}^{2}$ denote the informativeness, mean and variance of the point *q*, respectively, while α and β represent important factors for mean and variance, respectively, and determine the weights given to mean and variance to calculate the informativeness of point *q*. The adaptive sampling strategy then optimizes the path (next waypoint) for the robot,

(7) $$x^{*}\left(t+1\right)=\arg\max_{q\in Q_{*}\left(t\right)}I\left(q\right).$$

In [33], the bounds of the β parameter were presented in the context of the probability of achieving no regrets in the information gain. It is trivial to observe that low β can bias toward exploitation and high β can bias toward exploration. For instance, it was found that β should be proportional to the time instant for achieving minimum cumulative regret (mean value of the function in Equation (1)), defined as $R_{T}=\sum_{1}^{T}r_{t}=\sum_{1}^{T}f\left(x^{*}\right)-f\left(x_{t}\right)$, where $x_{t}$ is the location chosen by the path planning objective in Equation (7) at instant *t*. However, we study how these bounds are connected to the robot’s sampling objectives of both exploration and exploitation (maximizing mean and minimizing variance to obtain an accurate and fully known map of the function under study) as well as to consider its limited resources (energy, time, etc.). Therefore, in our work, we set a constraint that α+β=1 to use a singular parameter in analyzing the influence of the adaptive sampling method on several metrics. In contrast to [33], α=1, and β is a time-varying parameter, which we will show in the later sections is not optimal for reducing the overall mapping uncertainty. Based on different weighted combinations of mean and variance in the informative function, we used the following approaches:

1. MaxMean—The MaxMean approach chooses the point with maximum intensity value, i.e., max mean location as the next sampling point.
2. Alpha0.75—The Alpha0.75 approach selects the location with the highest information value given α=0.75.
3. Alpha0.5—The Alpha0.5 method selects the location with the highest information value given α=0.5.
4. Alpha0.25—The Alpha0.25 approach selects the location with the highest information value given α=0.25.
5. MaxVar—The MaxVar approach chooses the location with the lowest confidence value as the target location.
6. MaxVarMaxMean—The MaxVarMaxMean approach first selects the points with maximum uncertainty until a given threshold for confidence (variance) is reached. After satisfying the threshold, it then selects the points with MaxMean as the point of interest.

Table 2 enlists the values of alpha and beta for different variants used in this study. As per Table 1, most utility functions in informative sampling rely on the mean, variance, or some combination of the two. Because of diversity, we have not used the same weights as in the literature but instead used a range of weights that can give a general idea of how a fixed increase in weights can affect mapping performance. Nevertheless, this analysis can also be used to shed light on a specific scenario of exploration and exploitation.

## 4. Experiment Design and Implementation

We developed the simulations using the Robot Operating Systems (ROS [34]) Gazebo simulation framework, built on top of the open-source code base from [21]. We considered a 10 m × 15 m simulated area free of obstacles (to avoid bias in the analysis due to collision-avoidance algorithms). Until the robot’s battery is depleted, it takes samples based on the informative function, navigates to the location, and collects samples. With each new sample, the Gaussian process regression is trained, the intensity values of the whole environment (map) are predicted, and the informativeness of each location is updated based on one of the six variants in Section 3, which includes six adaptive sampling variants and the two baseline non-adaptive sampling variants. The robot’s starting location and battery timing were kept fixed for all scenarios.

**Figure 1:**
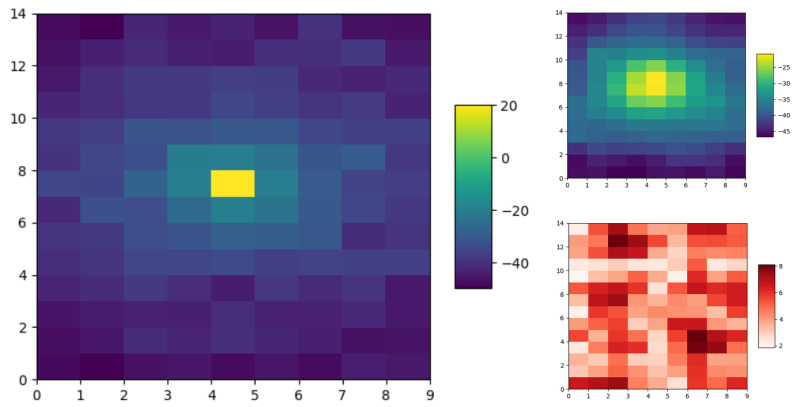
An image of the Wi-Fi signal ground truth (**left**) at source location (4, 7) and predicted GP mean (**top right**) and variance (**bottom right**) of a simple random walk exploration strategy.

The ground truth for the Wi-Fi signal map (Figure 1) was generated as per the equation for the received signal strength indicator (RSSI) [35,36]:

(8) $$RSSI=RSSI_{d0}-10\eta \ln\left(d\right)+\chi_{g},$$

where $RSSI_{d0}=TX_{power}-20\ln\left(\frac{3}{4\pi *f}\right)$ is the signal reference power at d0 = 1 m, *f* is the signal frequency (2.4 GHz), $TX_{power}$ is the power of the signal transmitter, η is the path loss factor (η=3), *d* is the distance between the signal source (i.e., an Access Point) and robot, $\chi_{g}$ is a Gaussian distribution with zero mean and variance (0.65 dB$m^{2}$) to represent noise in signals, similar to the settings in [21].

**Figure 2:**
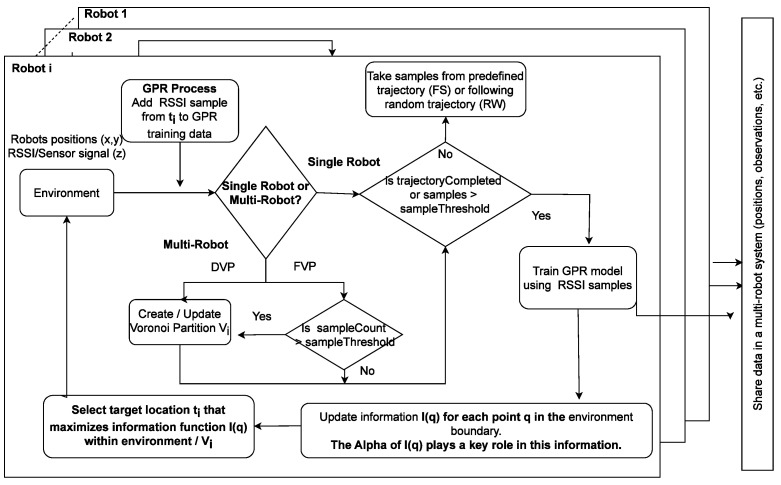
Block diagram of the distributed Gaussian process regression used in informative path planning.

Figure 2 presents an architectural overview of the control flow in the GPR-based informative path planning approach and the experimental design to analyze the influence of the information function (Equation (6)) on the outcome of mapping objectives.

### 4.1. Single-Robot Experiments

For single-robot experiments, we deployed a hector UAV robot (an aerial robot) with a battery capacity to sustain 500 ROS seconds (with a real-time factor close to 1) and a starting position of (4.5, 0). To provide a thorough analysis of the impact of exploration and exploitation on online learning and the mapping of the spatial distribution map, we consider two baseline scenarios:

1. Fixed Sweep (FS)
2. Random Walk (RW)

In a Fixed Sweep (FS) baseline, the UAV sweeps the whole region in a horizontal parallel strip pattern starting from the bottom center of the region to the upper center of the region. The idea is to make UAVs familiar with the overall intensity changes of the environment. In a random walk (RW) baseline, the UAV randomly explores the surrounding region after randomly moving up, down, left, or right by 3 points from its current position. All the α variants would use the RW as the initial approach for their minimum number of samples before using GPR and the Information function in Equation (6) to choose the next sampling point. Once the UAV finishes sweeping the whole region or has taken at least 15 samples from the surroundings (using RW), the informativeness of each location is updated, and the next sampling location is chosen based on the information function variant.

Following the ideas of the time-varying nature of the β coefficient in [33] and to further support our analysis, we include a new **TimeVariant (TW)** approach that dynamically varies the α value with respect to the time evolution of the mission, gradually moving from exploration (giving full priority to the uncertainty of the information value in Equation (6)) to exploitation (giving full priority to the predicted mean value of the information function). Inspired by a similar approach to balancing coverage and learning in [25] and balancing coverage and recharging in [24] throughout the mission duration, we present the time-variant coefficient below in Equation (9) that dynamically changes the priority from exploration to exploitation based on the expected mission period (e.g., based on the maximum energy capacity or the task requirement).

(9) $$\begin{array}{c} \alpha \left(t\right)=\left\{\begin{array}{cc} 0\;(\mathrm{Random}\;\mathrm{Walk}) & \mathrm{until}\;t_{min}\;\mathrm{to}\;\mathrm{collect}\;\min.\;\#\;\mathrm{of}\;\mathrm{samples}\\ \frac{t-t_{min}}{t_{max}} & \mathrm{at}\;\mathrm{any}\;\mathrm{time}\;t\geq t_{min}\\ 1 & \mathrm{at}\;t_{max}-\mathrm{end}\;\mathrm{of}\;\mathrm{mission}\;\mathrm{duration} \end{array}\right. \end{array}$$

Here, $t_{min}$ is the minimum time required to collect enough samples with which GPR can become useful, $t_{max}$ is the maximum time allocated for the mission. The idea for this time-varying α(t) is to generalize the dependence of α with respect to robot limitations such as energy, communication, and task requirements. For instance, energy-limited robots like UAVs can choose their α based on their current energy level. Higher energy means the robot can explore better, reaching farther regions, while lower energy can let it exploit the signal variations to find the peaks (sources).

This TimeVariant approach is expected to provide a balanced performance on multiple objectives in terms of mapping accuracy and source localization, but the cost of energy efficiency is not entirely known. Therefore, this paper will add this novel perspective in the comprehensive comparison of the sampling objectives when the information function priorities are fixed (a constant α in Table 2) or dynamically change during the mission (a time-variant α(t) in Equation (9)).

### 4.2. Multi-Robot Experiments

Researchers have used the Voronoi partitioning method in a multi-robot setting to divide an environment for multi-robot sampling [2,12,16]. Here, the robots are driven toward the centroids of the respective Voronoi region to maximize the mapping (sampling) performance and minimize the sensing cost. Robots choose the most informative location within the Voronoi region based on a utility function encompassing exploration and exploitation. Specifically, the work in [21] uses the heterogeneity of robots to weight the Voronoi partition, which is continually updated during the sampling process. Motivated by the investigations in the works mentioned above, we use Voronoi partitions to analyze multi-robot settings to distribute regions among multi-robots. To divide the given region $Q\subseteq R^{2}$ for n robots, we divide the environment into n regions that the partition $V_{i}$ for each robot that *i* corresponds to:

(10) $$V_{i}=\left\{q\in Q|∥q-p_{i}∥\leq ∥q-p_{j}∥,\forall j\neq i\right\}.$$

In the case of multi-robot sampling, we have considered the following two scenarios of Voronoi partitioning:

1. Fixed Voronoi Partition (FVP)—Considering only the initial robot positions, the region associated with the robot is decided at the start of the experiment. We fix these positions throughout the experiment, and the utility function determines what points within the respective region are to be chosen as target points.
2. Dynamic Voronoi Partition (DVP)—In this scenario, the Voronoi partition continuously updates as the robot moves. The target point can only be determined if it belongs to the respective partition at the time of the request based on the informative function.

For multi-robot experiments, 3 simulated Jackal UGV robots were deployed to the same Gazebo simulation framework. The initial positions of the three robots are (3, 2), (3, 10), and (7, 7), respectively. For the multi-robot sampling scenario, we only employed the RW baseline and took five random samples per robot (totaling 15 samples) within the Voronoi region before utilizing adaptive sampling. The baseline variant of both scenarios (FVP and DVP) is random walk sampling (non-adaptive).

### 4.3. Performance Metrics

We consider the following performance metrics:

1. Samples: The number of Wi-Fi signal strength samples the robot takes using its Wi-Fi device. This number should be minimized for better information sampling.
2. RMSE: The root mean squared error between the predicted mean information (Wi-Fi signal strength) through the GPR and the ground truth information. The aim is to obtain predictions as close as possible to the ground truth, i.e., lower RMSE. The RMSE values in the tables and figures represent the average RMSE over the whole map.
3. Variance: The confidence bounds of the predicted values given by the GPR. The goal is to be confident about the predicted mean value, i.e., lower variance. The variance values in the tables and figures represent the average variance over the entire map.
4. Cumulative Distance: Cumulative distance refers to the total distance traveled by the robot. The shorter the distance traveled, the lower the power consumption. We have used the cumulative distance metric to determine the energy cost incurred by the robot. The cumulative distance should be as low as possible for the optimum approach.
5. Source localization accuracy: If the location at where the maximum mean value of the predicted GP map lies within 1m of the actual source location, then that is classified as the correct localization; otherwise, the localization is incorrect. The localization accuracy is the percentage of correct localization of all trials out of all source location experiments combined.

The energy consumption of a robot depends mainly on two types of devices onboard a robot: time-dependent hardware resources (e.g., computer, controller, and sensors) and mobility-dependent hardware resources (e.g., motors and manipulators). Accordingly, we can derive the instantaneous change in the energy consumption equation of a mobile robot as [37],

(11) $$E_{i}\left(t\right)=E_{i}\left(t-1\right)-\alpha_{e}.dt-\beta_{e}.dx,$$

where $\alpha_{e}$ and $\beta_{e}$ are the coefficient parameters that provide importance to time-dependent and distance-dependent energy consumption. In real-world robotic systems, the motion (distance or velocity of the motors) significantly influences the energy characteristics of mobile robots [37,38]. For instance, it was found in [39] that the motion component consumes up to 95% of power in a mobile robot. Since we set the velocity of the robot constant in our analysis for the sake of controlled comparison of sampling objectives, we considered the *cumulative distance* as the key metric representing the energy consumption and obtained a measure of the energy efficiency of the robot’s sampling trajectory.

We ran five trials per variant in each scenario. Further, the experiments were repeated for five different Wi-Fi source locations, with each being at the middle, top-left, top-right, bottom-left, and bottom-right corners of the map area. In total, we conducted more than 1400 simulations for this analysis with the core results derived when the radio signal path loss factor in Equation (8) (η=3) and repeated them for a different loss factor (η=2) to analyze the impact of the environment in Section 5.4.

## 5. Results and Analysis

In adaptive sensing and the efficient modeling of environmental phenomena with robot-aided observations, the goal is to minimize the prediction (sensing) variance, improve the prediction (sensing) accuracy, and conserve energy by utilizing predictions promptly. It is generally understood that the exploration objective seeks to minimize the variance (uncertainty) of the predicted information, while exploitation seeks to minimize the RMSE of the predicted map (information accuracy). In both cases, we need to make use of the predictions as soon as possible. For instance, in the case of exploitation, we need to identify the signal source location, i.e., the place with the maximum signal intensity. An informative function can be either exploitation-based, exploration-based, or a weighted combination of both. In our variants, the MaxMean strategy does pure exploitation, whereas MaVar, Fixed Sweep, and Random Walk are pure exploration strategies. The rest of the variants combine exploration and exploitation. Below, we present the results of the single-robot and multi-robot experiments separately and then summarize the common analysis from an information sampling perspective.

### 5.1. Single-Robot Experiment Results

**Figure 3:**
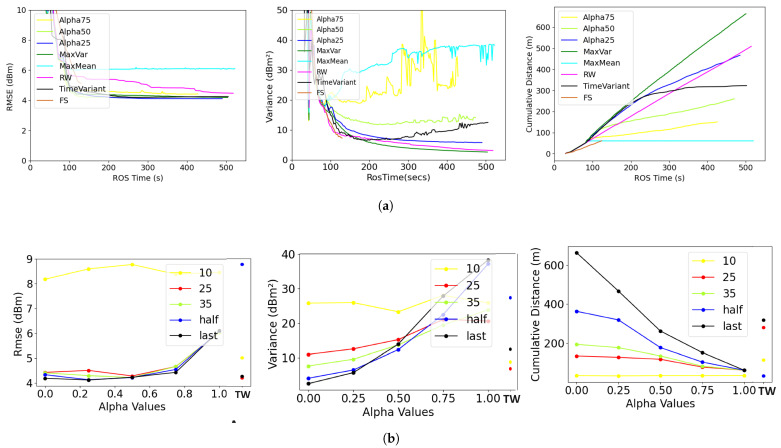
RMSE (**left**), variance (**center**), and cumulative distance (**right**) for different information functions in single-robot experiments. This figure is better visualized on a digital (colored) screen. (**a**) Single-robot experiment performance metrics over time; (**b**) single-robot experiment performance metrics over alpha.

**Figure 4:**
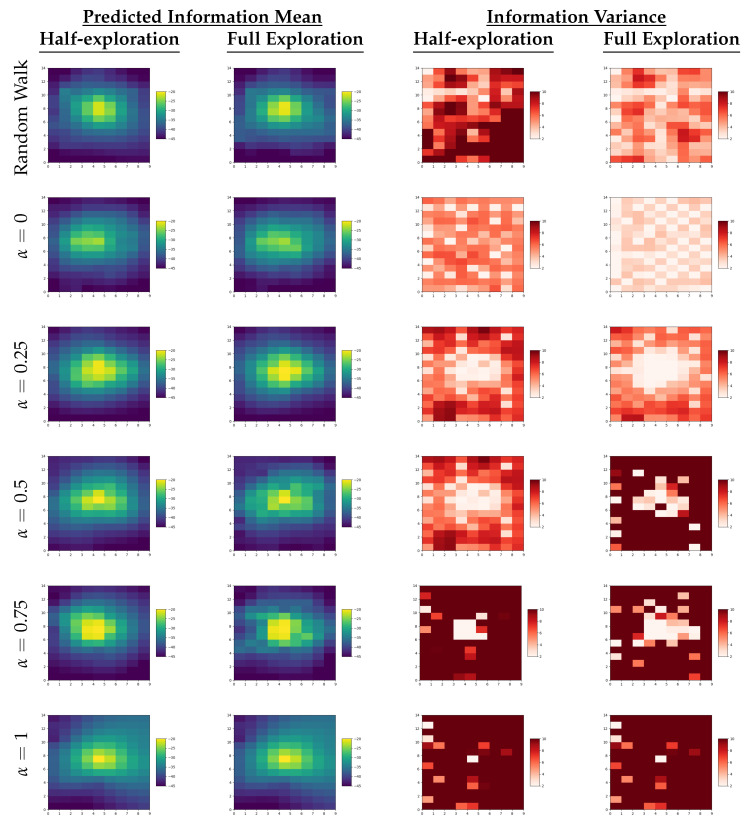
Predicted mean and uncertainty plots of the information function in the single-robot scenario, where the ground truth of the Wi-Fi signal distribution is depicted in Figure 1 with the source location at (4, 7).

Table 3 summarizes the performance metrics results obtained by averaging the data collected over all trials with different source locations for the single-robot experiments. Detailed results are shown in Figure 3, where the plots of performance metrics (RMSE, variance, and cumulative distance) are presented, comparing different variants of the analyzed information functions. An example evolution of the RMSE and variance of the GPR predictions for single-robot experiments can be seen in Figure 4.

In Figure 3b, summarized views of these performance metrics are presented from the perspective of the α parameter in different experiment settings. Here, we can visibly observe that the accuracy of the sensed information (RMSE) decreases when α and the uncertainty (variance) of the predicted information decreases with α. So, ideally, the α should be balanced to obtain high accuracy and low uncertainty. However, when the energy perspective is added (i.e., the cumulative distance), then the selection of α becomes complicated. Therefore, an in-depth discussion of this nature is essential to meaningfully analyze the tuning of the sampling function parameters in adaptive sensing and informative path planning applications. We present the discussion from the exploration and exploitation perspectives below. Depending on the mission requirements, one can choose the informative path-planning coefficients (α and β), and our study provided a direction toward this objective. It is worth noting that our focus lies on methodologies that accomplish both exploration and exploitation objectives while also conserving energy.

#### 5.1.1. Exploration Perspective

The exploration objective is to obtain accurate predictions of the sampled environmental process with the highest confidence bounds, i.e., the lowest variance (uncertainty) in all map areas. We compare the performance metrics with respect to the variation in the α values in Equation (6) at different instances during the exploration task.

As shown in Table 3 and Figure 3b, the higher the alpha values, the lesser the cumulative distance will be. Furthermore, the number of samples required for RMSE and variance saturation also increases with alpha. MaxVar, i.e., α=0, yielded the best convergence results for all cases, but at the cost of increased distance and the number of samples that the robot had to collect. In the MaxVar approach, the robot prioritizes exploring locations with higher uncertainty that have not yet been visited. These locations could be situated at a considerable distance from the robot. On the other hand, the time-varying alpha (TW) approach kept increasing α behaving like a MaxVar-like variant in the beginning and a MaxMean-like variant in the end. Due to this time-varying nature, the uncertainty has a huge variability and was very high (close to 12.85 dBm${}^{2}$) in the end, which could not meet the exploration objectives of the mission.

For α≥0.5, the values for variance are not stable as the robot prioritizes exploitation, gets stuck in local optima, and keeps taking samples from the same location without exploring further; therefore, new sampling data do not consistently improve the map variance. Hence, α values near 0.25 demonstrate optimal convergence and a well-balanced performance across all metrics. This is attributed to the fact that, in selecting the next target location to visit, the robot assigns higher importance to variance with a factor of α=0.75. This approach enables the robot to explore while considering locations with maximum mean values, facilitating a better understanding of the source. For a more accurate selection of alpha values, the energy consumption and variance must be considered depending on the specific mapping scenario.

#### 5.1.2. Exploitation Perspective

We take the source localization example as the objective of exploitation in our work. To properly locate a source, a robot should detect and provide a GP map with maximum mean at a point within 1 m of the real source location in any direction. Table 4 shows the source localization accuracy of all variances in all scenarios based on the number of times the resultant GP map can be used to correctly identify the source locations at different instances in the sampling process.

It can be observed from Table 4 that the localization accuracy of each approach improved with increasing numbers of samples. We are interested in identifying approaches to obtain better results with fewer observations. The RW, MaxMean, and FS approaches did not perform well, especially in the early stages of the experiment. With the FS baseline, fewer measurements are taken, while with MaxMean, the measurements are taken repetitively at the same location (local maxima) since the information function (with α=1) only depends on the predicted GP mean. The RW approach had improved source localization performance, but it was slower than the other approaches. RW’s results were better after just 25 samples than FS’s after 35 samples. In all scenarios (except FS), the initial 15 samples were obtained through random walk. Except for MaxMean, FS, and RW, all variants demonstrated strong performance in source localization. This can be attributed to their limited exploration and lack of consideration for information. Counterintuitively, giving full priority to the predicted mean value of the information function (by setting α=0, the sampling function could not obtain the source’s peak since learning the process was compromised by not accounting for the uncertainty of the predictions.

MaxVar, however, does not represent a cost-effective approach since it involves very long distances. MaxVarMaxMean, Alpha0.25, Alpha0.5, TW, and Alpha0.75 exhibit quicker convergence and cost effectiveness. If the MaxVarMaxMean approach fails to meet the variance and RMSE thresholds, it behaves identically to MaxVar.

We discovered that the alpha range 0.2≤α≤0.5 works well for exploitation objectives when minimizing distance cost is the first priority (e.g., if energy availability is heavily limited [37]). However, the increased variance continues to be a concern as the alpha value increases. To further narrow down the selection for exploitation within this range, the need to maintain a threshold variance versus energy consumption needs to be considered. In particular, Alpha0.25 is most effective when a balanced tradeoff is necessary, especially in scenarios where source localization accuracy needs to be enhanced. Interestingly, in situations where cost is not a concern, the MaxVar approach proved to be the most effective in achieving both mapping accuracy and confidence in exploitation performance.

### 5.2. Multi-Robot Experiment Results

**Figure 5:**
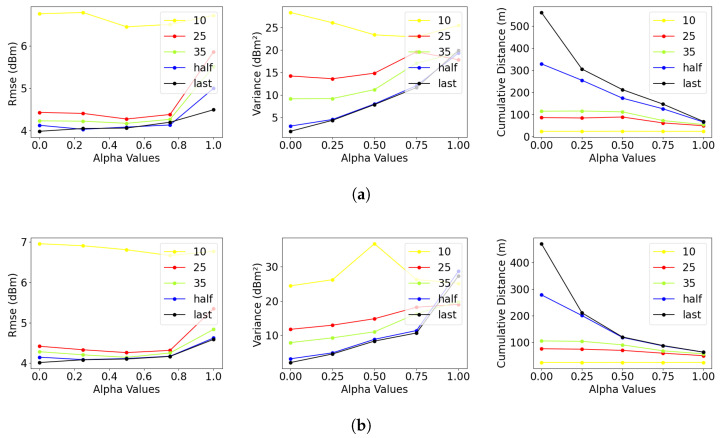
RMSE, variance, and cumulative distance for different alpha values and different sample counts in multi-robot experiments. This figure is better visualized on a digital (colored) screen. (**a**) Multi-robot fixed Voronoi partitioning (FVP) scenario; (**b**) multi-robot dynamic Voronoi partitioning (DVP) Scenario.

Table 5 summarizes the results for performance metrics obtained by averaging the data collected over all trials with different source locations for the multi-robot experiments. In Figure 5, summarized views of these performance metrics are presented from the perspective of the α parameter in different experiment settings. Similarly to the single-robot experiments, we can observe that the mapping accuracy (RMSE) and the uncertainty (variance) of the predicted information improve with the reduction in α. Accordingly, the α should be the lowest value for all robots in the team. But, as you can see in the MaxVar approach where α=0, the energy consumed is the highest. We discuss the impact of this parameter in a multi-robot setting below, with the aim of setting the α for all the robots to leverage the advantages offered by the multiple robots in completing the sampling mission.

#### 5.2.1. Exploration Perspective

The distance plots for multi-robot scenarios (see Figure 5a,b) show that all variants of DVP approaches had much shorter travel distances than the same approaches based on FVP. However, the FVP scenario resulted in improved variance as well as the speed of convergence compared to DVP scenarios. Consequently, we can conclude that the DVP scenario is suitable for cost-effective sampling (less energy), while the FVP scenario is suited for faster convergence and better exploration results. This is because, in the FVP approach, the robot is assigned to a fixed region, and its Voronoi partition does not change when it visits a corner. On the contrary, in DVP, when a robot visits a corner, its Voronoi partition undergoes a significant change from the previous time step, leading to longer distances.

Consistent with the findings in single-robot experiments, MaxVar has promising results in terms of variance and takes fewer samples, but its distance cost is almost twice that of Alpha0.75 and MaxVarMaxMean. Additionally, when α<0.5, we can observe stable variance with an extended distance. An α value close to 0.25 offers the optimal balance between all the performance metrics while performing close to the MaxVar approach. It effectively reduces variance and RMSE while keeping the increase in distance within an acceptable range.

#### 5.2.2. Exploitation Perspective

Table 6 presents the source localization results for the multi-robot experiments. Here, we observe similar results for both the multi-robot partitioning settings (FVP and DVP), where the Alpha0.25 variant still balances both source localization accuracy and the energy-consumption requirements well. After 25 samples, the dynamic and fixed Voronoi partitions performed close to each other, and they were successful at locating the source much faster, even with just 25 samples. This is expected, as more robots in the multi-robot system contribute to the task objectives and improve performance and efficiency. The performance improvements found with MaxMean and RW were lower than those of single-robot experiments. Generally, the alpha range 0.2≤α≤0.5 is useful to obtain a balanced performance for the exploitation objective. Higher Alpha variants like Alpha0.75 travel a lower distance and can localize the source faster but at the expense of decreased variance (see Table 5).

### 5.3. Impact of Source Locations on the Sampling Performance

We also analyzed the impact of different source locations on the sampling performance (results for these special cases are available in the Supplementary Materials). We found that there was almost no impact on the results across all sources, especially when the β value (i.e., the weights towards confidence bounds) was higher. However, for variants where α the value is higher (MaxMean, Alpha0.75, and Alpha0.5), they gave significantly different results for the furthest source locations at the bottom-right (0, 14) and top-right (9, 14) parts of the map area. This could be attributed to the fact that when α is higher, exploitation is more preferred, and therefore, localizing a much farther source could be difficult to accomplish. In summary, the effect of source locations was not observed for informative functions with greater weights for variance (exploration).

### 5.4. Impact of Wi-Fi Signal Distribution on the Sampling Performance

Further, we analyzed the impact of the signal distribution itself on the results by repeating all the single-robot cases with different path loss exponent (η=2 in Equation (8)) (results for these special cases are included in the Supplementary Materials). It was observed that all approaches with η = 2 performed quite well in comparison to the cases where η=3, and we found that the change in both variance and RMSE metrics was smoother for all variants when η = 2 than the same approaches when η = 3. Nevertheless, the change in the signal distribution had a minimal impact on our analysis, and the observations made for η=3 above hold for η=2 as well.

### 5.5. Summary of Findings

Our findings suggest that optimizing α can help strike a balance between the number of samples, the energy incurred, and the prediction accuracy while maintaining a high level of confidence. Specifically, assigning significance to the mean value is crucial. It is critical to give importance to both the mean and the variance of the predicted map; however, we determined that prioritizing the variance would quickly reduce the mapping uncertainty and help efficiently find the signal source in the map. This would allow the mapping process to simultaneously achieve exploration and exploitation objectives while maintaining a balance in the energy consumption attributed to the distance metric. Based on our analysis, an alpha value near 0.25 represents an optimal balance between the two objectives, enabling robots to model a physical process efficiently and accurately.

Our analysis can help to decide the α values for specific scenarios based on the objective. For instance, the objective outlined in [29] is for the robot to model the physical process, identify the source, and navigate to the source location. The proposed algorithm bears a resemblance to our MaxVarMaxMean approach. In this method, the algorithm explores the environment until a specific threshold is reached, subsequently employing MaxMean for exploitation. However, in real-world scenarios marked by noise and error-prone sensing, achieving the designated threshold for variance may be challenging for the robot. In such situations, continuous exploration persists, leading to increased distance traveled and higher energy consumption. On the other hand, a dynamic change in priority, as in the TimeVariant approach, could help balance the sampling objective (e.g., source localization) with other objectives such as achieving optimal coverage of the environment [25], but at the cost of increased uncertainty of the predicted data, which would be of extreme value in a mapping task.

To address this issue, the algorithm can be enhanced by incorporating Alpha0.25, which proved to provide the best balance of all metrics, including energy consumption. This modification assigns significance to both the mean and the variance throughout the experiment, demonstrating comparable performance to the MaxVar approach during exploration and effectively identifying the source location. Relaxing the threshold condition makes this approach less susceptible to variations in source location, ultimately conserving energy. Subsequently, the robot can navigate to the source location after reaching a predefined number of samples. A similar approach can be used in [16]. In methodologies similar to [11], the goal is to model the environment by utilizing an aerial robot and subsequently identifying the sources using a ground robot; Alpha0.75 proves to be a suitable choice in this context, mitigating energy consumption while maintaining a source localization accuracy on par with alternatives. However, it is worth noting that, as previously discussed, Alpha0.75 is accompanied by increased variance. Nevertheless, when it comes to applications safety-related applications like nuclear radiation mapping [40], where precision takes precedence over energy conservation, the MaxVar approach proves to be the most suitable choice.

Additional results are available as Supplementary Materials to this paper, which are available at https://hero.uga.edu/research/adaptivesampling/, accessed on 12 November 2023.

## 6. Conclusions

This study provided an understanding of balancing the exploration and exploitation tradeoff for different objectives by providing an in-depth analysis of various variants of informative functions in an adaptive sampling of the environmental phenomenon. Both the energy efficiency and mapping/localization objectives can be met with an optimum balance based on the specific objective and application domain. The analysis of our data and the results provide insights into choosing the best range of weight values for the exploration and exploitation coefficient. Our results show that α values near 0.25 provide the optimal balance for both exploration and exploitation objectives of sampling in scenarios where energy efficiency needs to be considered.

## Figures and Tables

**Table 1:** Comparison of the related works in the literature on adaptive information sampling. The Information functions used to obtain waypoints for successive sampling are adapted from the respective references.

Reference	Information Function	Scalability		Robot Type	Sampling Type	Objective	Property/Process Measured	Prediction Model	Exploration or Exploitation
		**Single Robot**	**Multi Robot**						
[1]	$I\left(P_{x,x^{\prime }}\right)=\frac{1}{\left|P_{x,x^{\prime }|}\right|}\sum_{\tau \in P_{x,x^{\prime }}}H\left(\tau \right)$	✓		Ground	Discrete	Mapping	Magnetic Field Intensity	Gaussian Processes	Exploration
[3]	$I_{i,j}=-\frac{\partial^{2}L\left(\theta_{m}\right)}{\partial \theta_{m,i,j}}$	✓	✓	Ground	Discrete	Mapping and source localization	Radio Signal Strength	Gaussian Processes	Mix
[4]	$I\left(Z_{A};Z_{B}\right)=I\left(Z_{B};Z_{A}\right)=H\left(Z_{A}\right)-H\left(Z_{A}|Z_{B}\right)$	✓		Marine	None	Monitoring	Salinity	Sparse Gaussian Processes	Exploration
[6]	$I^{rssi}\left(x\right)=\frac{max(\mu )-\mu *(x)}{max(\mu )-min(\mu )+\epsilon }$	✓		UGV	Continuous	Path Planning	Wi-Fi	Gaussian Processes	Exploitation
[10]	None	✓		Ground	Discrete (zigzag waypoints sweeping pattern)	Mapping	Gamma radiation	None	Exploration
[8]	$I\left(x\right)=\ln\left(\sigma \sqrt{2\pi e}\right)$	✓	✓	Ground	Discrete	Mapping	Radio Signal Strength	Gaussian Processes	Exploration
[11]	None		✓	Ground and Aerial	Continuous	Localization of Sources	Radiation	None	Exploration
[12]	$H\left[Y_{x_{i+1}}|d_{i}\right]=\log\sqrt{2\pi e\sigma_{Z_{x_{i+1}|d_{i}}}^{2}}+\mu_{Z_{x_{i+1}|d_{i}}}$		✓	Underwater	Discrete	Modeling	Algae	Gaussian Processes	Mix
[16]	$I\left(x\right)=\mu_{x|{\tilde{V}}_{i},y_{i}}^{*}+\beta \sigma_{x|{\tilde{V}}_{i},y_{i}}^{*2}$		✓	Ground	Discrete	Sensing Coverage	Stalk count, Temperature	Mixture of Gaussian Processes	Mix
[17]	None		✓	Ground and Aerial	Discrete	Mapping/Environmental Monitoring	Temperature, humidity, luminosity and carbon dioxide concentration	None	Exploration
[13]	None	✓		Ground	Discrete	Mapping	Radiation	None	Exploration
[18]	None	✓		Ground	Continuous	Localization of Source/Vehicle	Wi-Fi	Gaussian Processes	Exploration
[19]	$p\left(z_{newj}|x_{*}\right)=\Phi \left(\frac{z_{new,j}-E\left[z_{*j}\right]}{var\left(z_{*j}\right)}\right)$	✓		Ground	Discrete	Robots localization	Wireless signal strength	Path loss and Gaussian Processes	Exploration
[14]	None/$\varphi =\gamma_{d}+1/e^{{\beta_{0}}^{1/k}}$	✓		Ground	Discrete/Continuous	Mapping	Radiation	None	Exploration
[20]	$I:=abs\left(H\left(z_{1:t}\right)-H\left(z_{1:t}|x_{t}\right)\right)$	✓	None	Discrete	Localization of Hotspot		Radiation	None	Explore
[21]	$I\left(x\right)=\mu_{x|\bar{V},Y}^{*}+\beta \sigma_{x|\tilde{V},Y}^{{*}^{2}}$		✓	Ground and Aerial	Discrete	Mapping	Wi-Fi	Mixture of Gaussian Processes	Mix
[22]	None		✓	Ground	Continuous	Mapping	Gamma radiation	None	Exploration
[23]	None		✓	Ground and Aerial	Discrete/Continuous	Localization	Gamma radiation	None	Exploration

**Table 2:** Values of alpha and beta for different information function I(q) variants.

Approach	α	β	Inference
MaxMean	1	0	The predictive mean of the information will only be considered in choosing the next waypoint.
Alpha0.75	0.75	0.25	The prediction uncertainty of the information will be given more importance than the prediction mean in choosing the next waypoint.
Alpha0.5	0.5	0.5	The predictive mean and uncertainty will be equally considered in choosing the next waypoint.
Alpha0.25	0.25	0.75	The predictive mean of the information will be given more importance than the prediction uncertainty in choosing the next waypoint.
MaxVar	0	1	The prediction uncertainty of the information will only be considered in choosing the next waypoint.

**Table 3:** Single-robot experiment results: Mapping performance (mean and std) of the informative sampling functions with fixed sweep (FS) and random walk (RW) as the baselines in single-robot experiments. Here, the highlighted values in **boldface** indicate superior performance for specific metrics, while the bold values marked with a * correspond to the approach that offers the best balance.

Scenario	Samples (#)	RMSE (dBm)	Variance (dbm${}^{2}$)	Cumulative Distance (m)
Alpha0.75	205 ± 14.66	4.33 ± 0.35	26.27 ± 14.27	175.2 ± 75.87
Alpha0.5	194 ± 12.61	4.23 ± 0.1	16.13 ± 10.27	284.8 ± 91.59
Alpha0.25 *	168 ± 7.68 *	4.11 ± 0.07 *	5.73 ± 0.57 *	494.11 ± 70.57 *
MaxVar (α=0)	145 ± 3.46	4.18 ± 0.1	**2.49 ± 0.29**	696.28 ± 16.86
MaxMean (α=1)	226 ± 1	6.06 ± 1.1	37.84 ± 44.57	**61.44 ± 4.94**
MaxVarMaxMean	170 ± 10.63	4.2 ± 0.11	4.97 ± 0.54	489.65 ± 89.8
TimeVariant (TW)	189 ± 5.29	4.25 ± 0.17	12.85 ± 4.99	323.52 ± 34.59
FS	**36 ± 1**	4.82 ± 0.66	7.12 ± 2.13	68.32 ± 0.64
RW	150 ± 1	4.45 ± 0.22	3.1 ± 0.94	520.94 ± 6.34

**Table 4:** Source localization accuracy (%) of all approaches in the single-robot experiments. Here, the **bold** values signify the top-performing values (i.e., high accuracy with low samples), while the * values indicate the number of samples where localization accuracy was significantly high compared to previous values.

Samples	Alpha0.75	Alpha0.5	Alpha0.25	MaxVar	MaxMean	MaxVarMaxMean	TimeVariant	FS	RW
10	56	48	44	60	52	56	28	24	40
25 *	**96**	**96**	**96**	**100**	56	**100**	**96**	28	64
35	100	96	96	100	56	100	96	-	**80**
45	100	96	100	100	56	100	96	-	80
50	100	100	100	100	56	100	96	-	80
After half samples	100	100	100	100	56	100	100	48	80
After last sample	100	100	100	100	56	100	100	68	100

**Table 5:** Multi-robot experiment results: Mapping performance (mean and std) of the informative sampling functions with fixed Voronoi partitioning (FVP) and dynamic Voronoi partitioning (DVP) in multi-robot experiments. Here, the highlighted values in **boldface** indicate superior performance for specific metrics, while the bold values marked with a * correspond to the approach that offers the best balance.

FVP Scenario	Samples (#)	RMSE (dBm)	Variance (dbm${}^{2}$)	Cumulative Distance (m)
Alpha0.75	426 ± 34.81	4.11 ± 0.12	9.78 ± 2.82	149.12 ± 39.72
Alpha0.5	403 ± 35.76	4.05 ± 0.1	6.52 ± 1.69	224.74 ± 58.42
Alpha0.25 *	361 ± 31.51 *	4.02 ± 0.1 *	3.87 ± 0.68 *	332.02 ± 60.44 *
MaxVar (α=0)	236 ± 5	**4.02 ± 0.07**	**1.87 ± 0.13**	592.91 ± 8.69
MaxMean (α=1)	445 ± 44.78	4.97 ± 0.91	17.07 ± 5.91	**68.8 ± 16.94**
MaxVarMaxMean	377 ± 29.65	4.06 ± 0.08	4.2 ± 0.23	250.19 ± 28.6
RW	**204 ± 6.24**	4.77 ± 0.46	4.74 ± 1.3	514.62 ± 5.96
**DVP Scenario**	**Samples (#)**	**RMSE (dBm)**	**Variance (dbm** ${}^{2}$ **)**	**Cumulative Distance (m)**
Alpha0.75	463 ± 51.11	4.16 ± 0.15	10.1 ± 1.98	89.6 ± 16.35
Alpha0.5	408 ± 39.29	4.1 ± 0.1	8.06 ± 0.97	120.42 ± 11.84
Alpha0.25	382 ± 34.22 *	4.08 ± 0.1 *	4.5 ± 0.47 *	219.16 ± 29.98 *
MaxVar (α=0)	244 ± 10.72	**4.01 ± 0.07**	**1.86 ± 0.27**	538.42 ± 47.83
MaxMean (α=1)	444 ± 29.83	4.55 ± 0.55	24.08 ± 34.05	**62.94 ± 11**
MaxVarMaxMean	408 ± 37.96	4.07 ± 0.09	4.34 ± 0.27	233.89 ± 29.84
RW	**211 ± 20.95**	4.65 ± 0.33	3.71 ± 1.01	485.69 ± 72.39

**Table 6:** Source localization accuracy (%) for all variants in the multi-robot experiments using the FVP and DVP approaches. Here, the **bold** values signify the top-performing values, while the values with * indicate the number of samples where localization accuracy was significantly higher than in the previous row.

		Samples	Alpha0.75	Alpha0.5	Alpha0.25	MaxVar	MaxMean	MaxVarMaxMean	RW
**Multi-robot cases**	**FVP**	10	40	40	32	36	36	48	48
		25 *	**96**	**100**	**96**	**96**	44	**100**	64
		**35**	**100**	**100**	**100**	**100**	56	**100**	64
		45	100	100	100	100	64	96	60
		50	100	100	100	100	64	96	60
		After half sample	100	100	100	100	76	100	60
		After last sample	100	100	100	100	76	100	60
	**DVP**	10	36	40	28	32	32	40	48
		25 *	**100**	**100**	**100**	**92**	56	**88**	56
		**35**	**100**	**100**	**100**	**96**	76	**100**	56
		45	100	100	100	96	88	100	60
		50	100	100	100	96	92	100	60
		After half samples	100	100	100	100	96	100	60
		After last sample	100	100	100	100	96	100	68

## Data Availability

No new data were created or analyzed in this study. Data sharing is not applicable to this article.

## Supplementary Materials

The following supporting information can be downloaded at: https://www.mdpi.com/article/10.3390/s23239600/s1.

## Abbreviations

The following abbreviations are used in this manuscript:

GP	Gaussian Process
GPR	Gaussian Process Regression
GPS	Global Positioning System
ROS	Robot Operating System
RW	Random Walk
FS	Fixed Sweep
FVP	Fixed Voronoi Partition
DVP	Dynamic Voronoi Partition
RRT	Rapidly exploring random tree
RMSE	Root Mean Square Error
RSSI	Received Signal Strength Indicator
