# Supplementary material for: Exploration–Exploitation Tradeoff in the Adaptive Information Sampling of Unknown Spatial Fields with Mobile Robots
Source: Sensors (Basel). 2023 Dec 4;23(23):9600. doi: 10.3390/s23239600 (PMC10708738; doi:10.3390/s23239600)
Supplement: Supplementary file 1 [file sensors-23-09600-s001.zip › sensors-2714161-supplementary.pdf]

Analysis of Exploration vs. Exploitation in Adaptive Information Sampling

Appendix SA - Results with Extended Data

TABLE SI

PERFORMANCE METRICS FOR VARIANTS WITH INITIAL FIXED SWEEP SCENARIO WITH SIGNAL PROPAGATION EXPONENT N=3.

Approach	Source location	Samples	Rmse	Variance	Cumulative Distance
Alpha 0.75	**Src(0,0)**	**218 ± 3.46**	**4.3 ± 0.07**	**8.14 ± 2.5**	**87.2 ± 1.31**
	Src(0,14)	219 ± 4	4.08 ± 0.05	16.55 ± 16.73	97.03 ± 21.16
	Src(4,7)	222 ± 0	4.38 ± 0.21	9.25 ± 1.16	79.65 ± 1.55
	**Src(9,0)**	**220 ± 1**	**4.29 ± 0.1**	**7.1 ± 1.14**	**83.86 ± 2.27**
	Src(9,14)	216 ± 5.48	4.32 ± 0.09	15.09 ± 11.41	118.02 ± 43.35
Alpha 0.5	**Src(0,0)**	**212 ± 4.8**	**4.28 ± 0.06**	**9.62 ± 3.2**	**143.65 ± 35.3**
	Src(0,14)	204 ± 8.72	4.1 ± 0.08	21.44 ± 15.62	183.58 ± 67.3
	Src(4,7)	198 ± 6.08	4.22 ± 0.05	12.25 ± 5.16	227.79 ± 44.2
	**Src(9,0)**	**210 ± 6.71**	**4.17 ± 0.06**	**14.83 ± 14.58**	**139.26 ± 45.71**
	Src(9,14)	213 ± 6.56	4.2 ± 0.08	9.1 ± 2.81	147.36 ± 53.53
Alpha 0.25	**Src(0,0)**	**188 ± 5.66**	**4.17 ± 0.07**	**5.67 ± 0.46**	**320.34 ± 53.94**
	**Src(0,14)**	**184 ± 5.2**	**4.06 ± 0.08**	**5.67 ± 0.11**	**325.42 ± 35.96**
	Src(4,7)	174 ± 0	4.14 ± 0.07	4.73 ± 0.11	434.77 ± 10.35
	Src(9,0)	182 ± 6.56	4.16 ± 0.04	5.87 ± 0.32	362.33 ± 55.26
	Src(9,14)	182 ± 7.55	4.1 ± 0.08	5.87 ± 0.28	386.88 ± 61.49
Max Variance	Src(0,0)	153 ± 2.45	4.18 ± 0.03	2.57 ± 0.1	617.44 ± 18.47
	**Src(0,14)**	**153 ± 2**	**3.95 ± 0.08**	**2.44 ± 0.17**	**612.12 ± 14.42**
	Src(4,7)	155 ± 0	4.44 ± 0.13	3.0 ± 0.11	618.93 ± 15.65
	Src(9,0)	154 ± 1.73	4.2 ± 0.04	2.17 ± 0.18	625.74 ± 14.94
	**Src(9,14)**	**153 ± 2.65**	**4.16 ± 0.06**	**2.23 ± 0.14**	**617.44 ± 17.19**
Max Mean	Src(0,0)	221 ± 0	4.31 ± 0.11	11.39 ± 6.55	84.99 ± 0.14
	Src(0,14)	222 ± 0	4.48 ± 0.23	69.25 ± 73.97	78.95 ± 1.06
	**Src(4,7)**	**222 ± 1**	**4.77 ± 0.23**	**8.81 ± 0.63**	**77.74 ± 0.59**
	**Src(9,0)**	**222 ± 0**	**5.51 ± 0.43**	**4.74 ± 0.38**	**81.08 ± 1.44**
	Src(9,14)	223 ± 0	4.49 ± 0.15	66.27 ± 33.04	70.39 ± 1.04
MaxVarMaxMean	**Src(0,0)**	**178 ± 15.68**	**4.28 ± 0.03**	**5.07 ± 0.37**	**402.33 ± 98.39**
	Src(0,14)	178 ± 8.06	4.06 ± 0.13	5.19 ± 0.38	431.82 ± 59.98
	Src(4,7)	161 ± 13.08	4.46 ± 0.07	3.76 ± 0.84	563.6 ± 102.96
	**Src(9,0)**	**180 ± 12.21**	**4.16 ± 0.1**	**5.14 ± 0.59**	**405.4 ± 80.93**
	Src(9,14)	190 ± 9.9	4.23 ± 0.13	5.46 ± 1.26	324.07 ± 80.26
Fixed Sweep	**Src(0,0)**	**36 ± 1.41**	**4.37 ± 0.07**	**6.84 ± 0.3**	**68.48 ± 0.74**
	Src(0,14)	36 ± 1	4.25 ± 0.12	8.16 ± 0.34	68.08 ± 0.24
	Src(4,7)	36 ± 1.41	5.07 ± 0.13	9.43 ± 1.96	68.72 ± 1.03
	Src(9,0)	36 ± 0	5.99 ± 0.09	3.75 ± 0.32	68.25 ± 0.24
	**Src(9,14)**	**37 ± 0**	**4.39 ± 0.04**	**7.43 ± 0.79**	**68.07 ± 0.1**

TABLE SII

PERFORMANCE METRICS FOR VARIANTS WITH INITIAL RANDOM WALK SCENARIO WITH SIGNAL PROPAGATION EXPONENT N=3.

Approach	Source location	Samples	Rmse	Variance	Cumulative Distance
Alpha 0.75	**Src(0,0)**	**206 ± 14.56**	**4.32 ± 0.04**	**23.98 ± 10.6**	**140.94 ± 75.37**
	Src(0,14)	191 ± 16.76	4.11 ± 0.12	18.94 ± 8.66	235.2 ± 63.26
	**Src(4,7)**	**207 ± 12.57**	**4.38 ± 0.2**	**25.1 ± 3.64**	**197.24 ± 92.54**
	Src(9,0)	209 ± 10.49	4.23 ± 0.05	27.19 ± 12.45	143.65 ± 29.05
	Src(9,14)	213 ± 6.56	4.59 ± 0.64	36.14 ± 22.46	158.95 ± 56.36
Alpha 0.5	**Src(0,0)**	**201 ± 6.32**	**4.24 ± 0.06**	**15.19 ± 1.78**	**234.19 ± 42.78**
	Src(0,14)	198 ± 7.62	4.11 ± 0.05	23.0 ± 20.5	265.11 ± 57.2
	Src(4,7)	181 ± 6.32	4.32 ± 0.07	13.37 ± 4.37	392.72 ± 51.95
	**Src(9,0)**	**196 ± 11.45**	**4.22 ± 0.09**	**14.82 ± 3.07**	**260.51 ± 92.4**
	Src(9,14)	194 ± 16.82	4.24 ± 0.07	14.24 ± 3.76	271.49 ± 101.21
Alpha 0.25	Src(0,0)	164 ± 7.62	4.15 ± 0.07	6.03 ± 0.6	530.88 ± 44.93
	**Src(0,14)**	**169 ± 4.47**	**4.03 ± 0.03**	**5.89 ± 0.35**	**466.23 ± 57.16**
	Src(4,7)	164 ± 2.24	4.1 ± 0.08	4.84 ± 0.1	535.12 ± 22.62
	Src(9,0)	172 ± 11.4	4.15 ± 0.06	5.94 ± 0.33	467.92 ± 109.09
	**Src(9,14)**	**169 ± 5.66**	**4.12 ± 0.04**	**5.94 ± 0.2**	**470.4 ± 46.25**
Max Variance	**Src(0,0)**	**146 ± 3.32**	**4.21 ± 0.06**	**2.43 ± 0.09**	**685.97 ± 22.06**
	**Src(0,14)**	**145 ± 3.61**	**4.07 ± 0.09**	**2.39 ± 0.16**	**686.95 ± 12.98**
	Src(4,7)	145 ± 3.87	4.25 ± 0.08	2.99 ± 0.13	703.79 ± 10.7
	Src(9,0)	146 ± 3.74	4.19 ± 0.07	2.26 ± 0.19	700.75 ± 8.85
	Src(9,14)	145 ± 3	4.16 ± 0.09	2.36 ± 0.08	703.92 ± 15.65
Max Mean	Src(0,0)	226 ± 0	4.88 ± 0.11	76.42 ± 50.15	57.99 ± 1.27
	Src(0,14)	225 ± 1.41	6.05 ± 1.42	62.87 ± 61.2	65.17 ± 8.5
	Src(4,7)	226 ± 1	6.23 ± 0.58	25.7 ± 3.18	58.29 ± 0.4
	**Src(9,0)**	**226 ± 0**	**7.05 ± 0.52**	**11.67 ± 7.62**	**62.5 ± 2.35**
	**Src(9,14)**	**226 ± 0**	**6.12 ± 0.99**	**12.53 ± 4.21**	**63.27 ± 1.45**
MaxVarMaxMean	Src(0,0)	170 ± 11.05	4.26 ± 0.06	4.84 ± 0.11	489.43 ± 58.39
	**Src(0,14)**	**173 ± 5.48**	**4.09 ± 0.07**	**5.1 ± 0.18**	**459.19 ± 62.19**
	Src(4,7)	160 ± 3.87	4.26 ± 0.08	5.23 ± 1.12	586.45 ± 38.89
	Src(9,0)	169 ± 9.54	4.27 ± 0.07	4.86 ± 0.17	506.21 ± 86.01
	**Src(9,14)**	**180 ± 10.3**	**4.12 ± 0.12**	**4.82 ± 0.15**	**406.98 ± 81.99**
Random Walk	**Src(0,0)**	**150 ± 1.41**	**4.52 ± 0.19**	**2.64 ± 0.3**	**520.5 ± 8.52**
	**Src(0,14)**	**150 ± 0**	**4.45 ± 0.1**	**2.52 ± 0.19**	**523.68 ± 2.16**
	Src(4,7)	149 ± 1.41	4.18 ± 0.04	4.91 ± 0.15	520.63 ± 6.13
	Src(9,0)	150 ± 1	4.6 ± 0.23	2.81 ± 0.33	522.13 ± 4.16
	Src(9,14)	150 ± 1.41	4.5 ± 0.21	2.62 ± 0.27	517.76 ± 7.03

TABLE SIII

PERFORMANCE METRICS FOR VARIANTS WITH INITIAL FIXED SWEEP SCENARIO WITH SIGNAL PROPAGATION EXPONENT N=2.

Approach	Source location	Samples	Rmse	Variance	Cumulative Distance
Alpha 0.75	Src(0,0)	218 ± 2	4.15 ± 0.13	7.04 ± 1.96	96.06 ± 10.12
	Src(0,14)	221 ± 1	4.05 ± 0.1	5.93 ± 1.93	81.12 ± 1.74
	Src(4,7)	221 ± 1	4.3 ± 0.11	6.61 ± 1.56	77.77 ± 0.44
	Src(9,0)	218 ± 3.16	4.24 ± 0.09	6.92 ± 2.88	108.05 ± 25.1
	Src(9,14)	222 ± 0	4.18 ± 0.05	4.48 ± 1.09	69.57 ± 0.7
Alpha 0.5	Src(0,0)	211 ± 2.65	4.21 ± 0.06	6.1 ± 0.43	128.74 ± 8.37
	Src(0,14)	214 ± 6.32	4.05 ± 0.14	5.53 ± 2.61	122.34 ± 51.18
	Src(4,7)	208 ± 6.71	4.17 ± 0.05	4.89 ± 0.43	166.94 ± 39.86
	Src(9,0)	216 ± 2.24	4.13 ± 0.04	4.9 ± 0.8	108.15 ± 6.37
	Src(9,14)	218 ± 4.58	4.24 ± 0.11	6.35 ± 2.15	109.94 ± 37.61
Alpha 0.25	Src(0,0)	197 ± 3.61	4.03 ± 0.05	3.8 ± 0.21	244.17 ± 34.61
	Src(0,14)	183 ± 5.83	3.94 ± 0.05	3.99 ± 0.15	325.84 ± 52.99
	Src(4,7)	180 ± 3.74	4.03 ± 0.04	3.15 ± 0.1	384.61 ± 36.33
	Src(9,0)	197 ± 9.49	4.1 ± 0.07	3.77 ± 0.16	238.37 ± 68.86
	Src(9,14)	184 ± 7.87	4.11 ± 0.05	3.91 ± 0.9	358.96 ± 69.58
Max Variance	Src(0,0)	154 ± 1.41	4.07 ± 0.08	1.6 ± 0.13	629.57 ± 8.02
	Src(0,14)	151 ± 1	3.96 ± 0.04	1.54 ± 0.13	617.18 ± 21.43
	Src(4,7)	150 ± 4.9	4.16 ± 0.05	1.95 ± 0.2	626.02 ± 26.1
	Src(9,0)	151 ± 2.24	4.15 ± 0.02	1.68 ± 0.04	632.61 ± 9.79
	Src(9,14)	151 ± 4.47	4.18 ± 0.07	1.43 ± 0.21	620.46 ± 23.43
Max Mean	Src(0,0)	219 ± 1	4.21 ± 0.19	42.41 ± 72.38	86.39 ± 2.04
	Src(0,14)	221 ± 0	4.03 ± 0.02	5.69 ± 1.35	79.9 ± 1.79
	Src(4,7)	221 ± 0	4.39 ± 0.16	5.4 ± 0.41	77.83 ± 0.84
	Src(9,0)	221 ± 0	5.18 ± 0.03	2.84 ± 0.22	79.99 ± 0.23
	Src(9,14)	223 ± 0	4.27 ± 0.12	6.67 ± 1.62	71.31 ± 3.48
MaxVarMaxMean	Src(0,0)	213 ± 4.12	4.14 ± 0.13	4.49 ± 0.24	143.54 ± 25.95
	Src(0,14)	207 ± 11.66	4.06 ± 0.09	4.24 ± 0.33	193.91 ± 92.48
	Src(4,7)	214 ± 1.41	4.34 ± 0.09	4.62 ± 0.26	141.27 ± 16.72
	Src(9,0)	198 ± 8.19	4.19 ± 0.05	4.71 ± 0.22	266.91 ± 74.04
	Src(9,14)	213 ± 7	4.15 ± 0.11	3.8 ± 0.59	144.14 ± 38.94
Fixed Sweep	Src(0,0)	36 ± 1	4.28 ± 0.06	5.36 ± 0.76	68.19 ± 0.16
	Src(0,14)	36 ± 0	4.17 ± 0.15	3.8 ± 0.44	68.03 ± 0.13
	Src(4,7)	36 ± 1	4.69 ± 0.28	4.87 ± 0.67	68.27 ± 0.14
	Src(9,0)	37 ± 0	5.35 ± 0.15	3.13 ± 0.66	68.09 ± 0.33
	Src(9,14)	37 ± 0	4.28 ± 0.13	4.6 ± 0.51	68.2 ± 0.18

TABLE SIV

PERFORMANCE METRICS FOR VARIANTS WITH INITIAL RANDOM WALK SCENARIO WITH SIGNAL PROPAGATION EXPONENT N=2.

Approach	Source location	Samples	Rmse	Variance	Cumulative Distance
Alpha 0.75	Src(0,0)	199 ± 13.3	4.27 ± 0.19	68.25 ± 112.89	196.9 ± 50.99
	Src(0,14)	220 ± 7.94	3.98 ± 0.09	13.05 ± 4.11	100.6 ± 36.8
	Src(4,7)	218 ± 6.56	4.38 ± 0.2	14.56 ± 2.95	115.08 ± 47.51
	Src(9,0)	210 ± 10.54	4.21 ± 0.09	10.85 ± 2.14	169.41 ± 81.29
	Src(9,14)	223 ± 3.16	4.25 ± 0.1	9.01 ± 0.91	81.23 ± 20.35
Alpha 0.5	Src(0,0)	188 ± 17.69	4.11 ± 0.11	10.27 ± 0.44	337.03 ± 134.12
	Src(0,14)	194 ± 19.85	4.0 ± 0.11	8.15 ± 1.14	251.15 ± 117.29
	Src(4,7)	198 ± 4.24	4.23 ± 0.09	8.27 ± 3.82	248.43 ± 25
	Src(9,0)	205 ± 6.48	4.13 ± 0.12	8.56 ± 0.92	211.47 ± 53.82
	Src(9,14)	196 ± 15	4.1 ± 0.08	9.22 ± 2.33	279.89 ± 126.59
Alpha 0.25	Src(0,0)	170 ± 11.05	4.03 ± 0.03	4.22 ± 0.16	452.7 ± 58.65
	Src(0,14)	163 ± 10.25	3.99 ± 0.06	3.95 ± 0.39	499.61 ± 91.77
	Src(4,7)	166 ± 3.32	4.07 ± 0.06	3.21 ± 0.16	513.1 ± 30.55
	Src(9,0)	173 ± 9.22	4.11 ± 0.06	4.03 ± 0.06	432.53 ± 64.13
	Src(9,14)	179 ± 9.85	4.1 ± 0.06	3.84 ± 0.21	409.81 ± 81.41
Max Variance	Src(0,0)	145 ± 2.83	4.04 ± 0.07	1.71 ± 0.19	706.65 ± 20.44
	Src(0,14)	145 ± 1.41	4.0 ± 0.07	1.5 ± 0.14	714.51 ± 8.47
	Src(4,7)	142 ± 2	4.15 ± 0.07	1.88 ± 0.1	718.65 ± 25.38
	Src(9,0)	141 ± 5.83	4.17 ± 0.1	1.77 ± 0.17	709.49 ± 16.47
	Src(9,14)	141 ± 3.87	4.09 ± 0.09	1.64 ± 0.13	708.95 ± 12.11
Max Mean	Src(0,0)	218 ± 3.74	4.67 ± 0.41	310.41 ± 155.91	102.49 ± 23.41
	Src(0,14)	227 ± 0	4.43 ± 0.34	17.99 ± 4.97	60.72 ± 0.92
	Src(4,7)	223 ± 2.24	5.98 ± 1.47	126.23 ± 117.88	79.15 ± 17.39
	Src(9,0)	223 ± 3.46	5.46 ± 1.05	33.07 ± 55.04	84.8 ± 31.34
	Src(9,14)	226 ± 1	5.32 ± 0.91	6.37 ± 3.26	63.38 ± 2.25
MaxVarMaxMean	Src(0,0)	192 ± 4.58	4.03 ± 0.06	4.87 ± 0.11	322.8 ± 37
	Src(0,14)	171 ± 23.96	4.03 ± 0.05	5.04 ± 1.19	464.8 ± 167.34
	Src(4,7)	183 ± 13.34	4.2 ± 0.09	4.84 ± 0.18	404.13 ± 109.22
	Src(9,0)	191 ± 6.63	4.12 ± 0.03	4.84 ± 0.14	324.8 ± 32.79
	Src(9,14)	201 ± 7.55	4.24 ± 0.07	4.55 ± 0.29	246.06 ± 48.97
Random Walk	Src(0,0)	149 ± 0	4.23 ± 0.18	1.83 ± 0.29	526.27 ± 3.57
	Src(0,14)	149 ± 0	4.22 ± 0.1	1.69 ± 0.22	524.69 ± 4.04
	Src(4,7)	149 ± 2	4.12 ± 0.06	2.6 ± 0.25	518.56 ± 4.87
	Src(9,0)	149 ± 0	4.39 ± 0.28	1.77 ± 0.26	518.21 ± 6.42
	Src(9,14)	150 ± 1	4.36 ± 0.19	1.59 ± 0.21	517.81 ± 6.98

TABLE SV

PERFORMANCE METRICS IN MULTI-ROBOT FIXED VORONOI PARTITION (FVP) SCENARIOS.

Approach	Source location	Samples	Rmse	Variance	Distance
Alpha0.75	**Src(0,0)**	**427 ± 26.32**	**4.15 ± 0.05**	**8.25 ± 1.06**	**147.61 ± 22.12**
	Src(0,14)	413 ± 42.11	3.96 ± 0.07	7.39 ± 0.82	207.91 ± 27.87
	Src(4,7)	443 ± 40.07	4.14 ± 0.16	14.36 ± 2.27	99.02 ± 8.41
	**Src(9,0)**	**423 ± 23.52**	**4.21 ± 0.07**	**8.71 ± 1.24**	**137.31 ± 15.42**
	Src(9,14)	426 ± 31.46	4.08 ± 0.04	10.19 ± 0.93	153.77 ± 13.46
Alpha0.5	**Src(0,0)**	**392 ± 11.49**	**4.17 ± 0.02**	**5.15 ± 0.37**	**226.44 ± 34.79**
	Src(0,14)	368 ± 14.66	3.96 ± 0.08	4.85 ± 0.67	316.08 ± 17.68
	Src(4,7)	419 ± 13.6	3.97 ± 0.06	9.11 ± 0.65	148.17 ± 16.5
	**Src(9,0)**	**426 ± 31.98**	**4.08 ± 0.1**	**6.0 ± 0.5**	**232.51 ± 15.78**
	Src(9,14)	410 ± 51.71	4.05 ± 0.04	7.49 ± 0.66	200.52 ± 12.75
Alpha0.25	**Src(0,0)**	**341 ± 7.55**	**4.14 ± 0.05**	**3.05 ± 0.23**	**396.0 ± 18.19**
	Src(0,14)	333 ± 5.74	3.87 ± 0.03	3.32 ± 0.3	404.58 ± 19.24
	Src(4,7)	400 ± 26.68	4.03 ± 0.09	4.47 ± 0.24	269.8 ± 21.18
	**Src(9,0)**	**359 ± 32.48**	**4.04 ± 0.04**	**3.88 ± 0.2**	**301.65 ± 30.46**
	Src(9,14)	373 ± 16.79	4.03 ± 0.04	4.64 ± 0.32	288.09 ± 10.16
MaxVar	**Src(0,0)**	**233 ± 4.36**	**4.07 ± 0.02**	**1.82 ± 0.12**	**591.97 ± 11.31**
	Src(0,14)	237 ± 4.47	3.9 ± 0.03	1.85 ± 0.1	589.39 ± 7.57
	Src(4,7)	241 ± 1.41	4.01 ± 0.05	2.04 ± 0.07	594.53 ± 4.32
	**Src(9,0)**	**235 ± 3.74**	**4.07 ± 0.04**	**1.81 ± 0.09**	**592.34 ± 10.7**
	Src(9,14)	233 ± 3.74	4.06 ± 0.03	1.84 ± 0.11	596.3 ± 5.61
MaxMean	**Src(0,0)**	**451 ± 28.58**	**5.47 ± 1.2**	**13.17 ± 4.77**	**62.97 ± 8.44**
	**Src(0,14)**	**495 ± 50.08**	**5.29 ± 1**	**10.44 ± 3.45**	**80.98 ± 10.35**
	Src(4,7)	438 ± 32.28	5.3 ± 0.72	24.68 ± 2.81	52.73 ± 2.59
	Src(9,0)	426 ± 26.65	4.48 ± 0.09	19.15 ± 1.68	58.08 ± 8.81
	Src(9,14)	418 ± 35.83	4.3 ± 0.06	17.92 ± 2.68	89.22 ± 14.13
MaxVarMaxMean	**Src(0,0)**	**385 ± 25.63**	**4.14 ± 0.05**	**4.12 ± 0.22**	**226.57 ± 17.21**
	Src(0,14)	367 ± 30.53	3.99 ± 0.05	4.15 ± 0.3	240.89 ± 19.71
	Src(4,7)	390 ± 33.78	4.05 ± 0.05	4.3 ± 0.17	265.15 ± 21.57
	**Src(9,0)**	**381 ± 30.4**	**4.1 ± 0.07**	**4.17 ± 0.24**	**244.34 ± 30.61**
	Src(9,14)	360 ± 10.34	4.01 ± 0.08	4.27 ± 0.16	274.0 ± 23.08
RW	**Src(0,0)**	**206 ± 7.68**	**4.73 ± 0.09**	**4.41 ± 0.49**	**513.45 ± 5.02**
	**Src(0,14)**	**208 ± 4.36**	**4.81 ± 0.21**	**4.36 ± 0.48**	**512.87 ± 7.79**
	Src(4,7)	201 ± 4.69	4.11 ± 0.08	6.94 ± 0.66	518.83 ± 2.46
	Src(9,0)	201 ± 4.12	4.89 ± 0.48	4.42 ± 0.88	515.89 ± 4.42
	Src(9,14)	205 ± 6.24	5.32 ± 0.14	3.58 ± 0.45	512.06 ± 6.01

TABLE SVI

PERFORMANCE METRICS FOR MULTI-ROBOT DYNAMIC VORONOI PARTITION (DVP) SCENARIOS.

Approach	Source location	Samples	Rmse	Variance	Distance
Alpha0.75	**Src(0,0)**	**440 ± 22.52**	**4.12 ± 0.02**	**8.75 ± 0.99**	**93.36 ± 14.12**
	Src(0,14)	459 ± 37.76	4.14 ± 0.12	10.49 ± 2.04	90.44 ± 16.39
	Src(4,7)	533 ± 44.47	4.05 ± 0.07	11.49 ± 2.64	87.69 ± 13.62
	**Src(9,0)**	**442 ± 32.88**	**4.13 ± 0.06**	**9.74 ± 1.53**	**90.04 ± 15.02**
	Src(9,14)	443 ± 42.84	4.34 ± 0.21	10.0 ± 1.09	86.46 ± 20.69
Alpha0.5	Src(0,0)	406 ± 40.61	4.17 ± 0.05	8.01 ± 0.81	118.03 ± 11.78
	Src(0,14)	414 ± 31.24	4.07 ± 0.09	8.28 ± 0.38	121.39 ± 14.43
	Src(4,7)	395 ± 37.07	3.98 ± 0.06	8.38 ± 1.18	123.42 ± 9.97
	Src(9,0)	420 ± 46.97	4.08 ± 0.04	7.79 ± 1.19	120.66 ± 13.28
	Src(9,14)	405 ± 33.91	4.18 ± 0.06	7.83 ± 0.91	118.63 ± 7.72
Alpha0.25	**Src(0,0)**	**401 ± 21.75**	**4.14 ± 0.09**	**4.56 ± 0.35**	**201.79 ± 32.02**
	Src(0,14)	370 ± 42	4.02 ± 0.08	4.53 ± 0.37	216.35 ± 20.26
	Src(4,7)	361 ± 27.51	3.97 ± 0.04	3.98 ± 0.28	241.62 ± 21.72
	**Src(9,0)**	**385 ± 19.05**	**4.11 ± 0.02**	**4.64 ± 0.53**	**214.91 ± 39.74**
	Src(9,14)	393 ± 37.32	4.15 ± 0.06	4.78 ± 0.33	221.14 ± 13.03
MaxVar	Src(0,0)	249 ± 5.92	4.05 ± 0.03	1.72 ± 0.09	564.67 ± 7.72
	**Src(0,14)**	**242 ± 10.54**	**3.94 ± 0.1**	**1.82 ± 0.28**	**514.68 ± 78.59**
	Src(4,7)	240 ± 17.35	4.0 ± 0.05	2.22 ± 0.27	521.85 ± 54.78
	**Src(9,0)**	**241 ± 7**	**4.06 ± 0.04**	**1.83 ± 0.13**	**547.96 ± 15.25**
	Src(9,14)	246 ± 5.57	3.98 ± 0.03	1.73 ± 0.13	542.95 ± 18.25
MaxMean	Src(0,0)	457 ± 18.25	4.72 ± 0.41	16.03 ± 2.46	57.65 ± 5.13
	**Src(0,14)**	**450 ± 18.95**	**4.15 ± 0.1**	**15.3 ± 1.68**	**75.75 ± 6.78**
	Src(4,7)	457 ± 32.82	5.32 ± 0.63	57.51 ± 66.1	48.65 ± 3.88
	Src(9,0)	427 ± 33.2	4.25 ± 0.09	17.42 ± 4.07	63.51 ± 5.8
	**Src(9,14)**	**430 ± 26.76**	**4.33 ± 0.11**	**14.11 ± 1.05**	**69.14 ± 7.03**
MaxVarMaxMean	**Src(0,0)**	**410 ± 16.76**	**4.15 ± 0.03**	**4.23 ± 0.23**	**226.57 ± 40.15**
	Src(0,14)	400 ± 44.33	3.97 ± 0.07	4.49 ± 0.27	241.57 ± 36.57
	Src(4,7)	389 ± 22.45	4.03 ± 0.09	4.5 ± 0.25	244.56 ± 16.75
	**Src(9,0)**	**412 ± 37.13**	**4.09 ± 0.07**	**4.18 ± 0.27**	**231.55 ± 15.98**
	Src(9,14)	430 ± 46.38	4.12 ± 0.03	4.31 ± 0.09	225.17 ± 25.66
RW	**Src(0,0)**	**204 ± 9.43**	**4.68 ± 0.07**	**3.18 ± 0.39**	**495.62 ± 27.34**
	**Src(0,14)**	**204 ± 6.32**	**4.86 ± 0.2**	**2.86 ± 0.22**	**488.74 ± 23.65**
	Src(4,7)	207 ± 11.96	4.03 ± 0.04	5.28 ± 0.35	509.44 ± 21.2
	Src(9,0)	217 ± 18.19	5.0 ± 0.29	3.22 ± 0.57	499.83 ± 13.28
	Src(9,14)	221 ± 36.63	4.7 ± 0.26	4.0 ± 0.81	434.82 ± 144.25

TABLE SVII

SUMMARY OF PERFORMANCE METRICS FOR INITIAL FIXED SWEEP SCENARIO WITH N=3.

FS Scenario n=3	Samples	RMSE	Variance	Cumulative Distance
Alpha75	219 ± 4	4.27 ± 0.15	11.23 ± 9.93	93.15 ± 25.59
**Alpha50**	**207 ± 8.66**	**4.19 ± 0.09**	**13.45 ± 10.97**	**168.33 ± 60.57**
**Alpha25**	**182 ± 7.42**	**4.13 ± 0.08**	**5.56 ± 0.51**	**365.95 ± 63.34**
MaxVar	153 ± 2	4.18 ± 0.17	2.48 ± 0.33	618.33 ± 16.78
MaxMean	222 ± 0	4.71 ± 0.49	32.09 ± 46.63	78.63 ± 4.9
MaxVarMaxMean	177 ± 15.39	4.24 ± 0.17	4.92 ± 0.97	425.45 ± 115.94
FS	36 ± 1	4.82 ± 0.66	7.12 ± 2.13	68.32 ± 0.64

TABLE SVIII

SUMMARY OF PERFORMANCE METRICS FOR INITIAL RANDOM WALK SCENARIO WITH N=3.

RW Scenario n=3	Samples	RMSE	Variance	Cumulative Distance
Alpha75	205 ± 14.66	4.33 ± 0.35	26.27 ± 14.27	175.2 ± 75.87
**Alpha50**	**194 ± 12.61**	**4.23 ± 0.1**	**16.13 ± 10.27**	**284.8 ± 91.59**
**Alpha25**	**168 ± 7.68**	**4.11 ± 0.07**	**5.73 ± 0.57**	**494.11 ± 70.57**
MaxVar	145 ± 3.46	4.18 ± 0.1	2.49 ± 0.29	696.28 ± 16.86
MaxMean	226 ± 1	6.06 ± 1.1	37.84 ± 44.57	61.44 ± 4.94
MaxVarMaxMean	170 ± 10.63	4.2 ± 0.11	4.97 ± 0.54	489.65 ± 89.8
RW	150 ± 1	4.45 ± 0.22	3.1 ± 0.94	520.94 ± 6.34

TABLE SIX

SUMMARY OF PERFORMANCE METRICS FOR INITIAL FIXED SWEEP SCENARIO WITH N=2.

FS Variants n=2	Samples	Rmse	Variance	Cumulative Distance
Alpha75	220 ± 2.45	4.18 ± 0.13	6.19 ± 2.19	86.51 ± 18.35
**Alpha50**	**213 ± 6**	**4.16 ± 0.11**	**5.55 ± 1.69**	**127.22 ± 40**
**Alpha25**	**188 ± 10.05**	**4.04 ± 0.08**	**3.72 ± 0.52**	**310.39 ± 80.73**
MaxVar	151 ± 3.46	4.1 ± 0.1	1.64 ± 0.24	625.17 ± 20.06
MaxMean	221 ± 1.41	4.42 ± 0.42	12.6 ± 35.67	79.09 ± 5.23
MaxVarMaxMean	209 ± 9.54	4.18 ± 0.14	4.37 ± 0.48	177.95 ± 75.3
FS	36 ± 1	4.55 ± 0.47	4.35 ± 1.01	68.16 ± 0.22

TABLE SX

SUMMARY OF PERFORMANCE METRICS FOR INITIAL RANDOM WALK SCENARIO WITH N=2.

RW Variants n=2	Samples	Rmse	Variance	Cumulative Distance
Alpha75	214 ± 12.37	4.22 ± 0.2	23.15 ± 55.38	132.64 ± 67.37
**Alpha50**	**196 ± 15.13**	**4.12 ± 0.13**	**8.89 ± 2.25**	**265.59 ± 109.57**
**Alpha25**	**170 ± 10.68**	**4.06 ± 0.07**	**3.85 ± 0.41**	**461.55 ± 79.04**
MaxVar	143 ± 4	4.09 ± 0.1	1.7 ± 0.2	711.65 ± 18.14
MaxMean	223 ± 3.87	5.17 ± 1.09	98.82 ± 145.73	78.11 ± 24.49
MaxVarMaxMean	188 ± 16.61	4.12 ± 0.11	4.83 ± 0.58	352.52 ± 120.86
RW	149 ± 1	4.26 ± 0.2	1.9 ± 0.44	521.11 ± 6.45

TABLE SXI

SUMMARY OF PERFORMANCE METRICS FOR MULTI-ROBOT FIXED VORONOI PARTITIONING CASES WITH N=3.

FVP Scenario	Samples	RMSE	Variance	Cumulative Distance
Alpha75	426 ± 34.81	4.11 ± 0.12	9.78 ± 2.82	149.12 ± 39.72
**Alpha50**	**403 ± 35.76**	**4.05 ± 0.1**	**6.52 ± 1.69**	**224.74 ± 58.42**
**Alpha25**	**361 ± 31.51**	**4.02 ± 0.1**	**3.87 ± 0.68**	**332.02 ± 60.44**
MaxVar	236 ± 5	4.02 ± 0.07	1.87 ± 0.13	592.91 ± 8.69
MaxMean	445 ± 44.78	4.97 ± 0.91	17.07 ± 5.91	68.8 ± 16.94
MaxVarMaxMean	377 ± 29.65	4.06 ± 0.08	4.2 ± 0.23	250.19 ± 28.6
RW	204 ± 6.24	4.77 ± 0.46	4.74 ± 1.3	514.62 ± 5.96

TABLE SXII

SUMMARY OF PERFORMANCE METRICS FOR MULTI-ROBOT DYNAMIC VORONOI PARTITIONING CASES WITH N=3.

DVP Scenario	Samples	RMSE	Variance	Cumulative Distance
Alpha75	463 ± 51.11	4.16 ± 0.15	10.1 ± 1.98	89.6 ± 16.35
**Alpha50**	**408 ± 39.29**	**4.1 ± 0.1**	**8.06 ± 0.97**	**120.42 ± 11.84**
**Alpha25**	**382 ± 34.22**	**4.08 ± 0.1**	**4.5 ± 0.47**	**219.16 ± 29.98**
MaxVar	244 ± 10.72	4.01 ± 0.07	1.86 ± 0.27	538.42 ± 47.83
MaxMean	444 ± 29.83	4.55 ± 0.55	24.08 ± 34.05	62.94 ± 11
MaxVarMaxMean	408 ± 37.96	4.07 ± 0.09	4.34 ± 0.27	233.89 ± 29.84
RW	211 ± 20.95	4.65 ± 0.39	3.71 ± 1.01	485.69 ± 72.39

TABLE SXIII

SOURCE LOCALIZATION MEAN ACCURACY (%) BY ALL APPROACHES.

		**Samples**	**Alpha75**	**Alpha50**	**Alpha25**	**MaxVar**	**MaxMean**	**MaxVarMaxMean**	**RW/FS**
**Singe robot cases**	**FS (n=2)**	10	28	24	24	20	20	24	20
		25	32	40	40	40	36	40	36
		**35**	**84**	72	72	68	68	64	
		**45**	**88**	**100**	**96**	**88**	**72**	**88**	
		50	88	100	96	100	72	92	
		After half samples	100	100	100	100	72	92	44
		After last sample	100	100	100	100	72	92	76
	**FS (n=3)**	10	24	32	28	28	32	28	24
		25	40	32	24	40	32	32	28
		**35**	**84**	68	68	72	68	76	
		**45**	**100**	**100**	**100**	**92**	**72**	**96**	
		50	100	100	100	92	72	92	
		After half samples	100	100	100	96	72	96	48
		After last sample	100	100	100	100	72	100	68
	**RW (n=2)**	10	40	32	44	44	32	44	32
		**25**	**96**	**100**	**100**	**100**	72	**100**	64
		**35**	**100**	**100**	**100**	**96**	72	**100**	68
		45	100	100	100	100	72	100	68
		50	100	100	96	100	72	100	72
		After half samples	100	100	100	100	72	100	76
		After last sample	100	100	100	100	72	100	100
	**RW (n=3)**	10	56	48	44	60	52	56	40
		**25**	**96**	**96**	**96**	**100**	56	**100**	64
		**35**	**100**	**96**	**96**	**100**	56	**100**	**80**
		45	100	96	100	100	56	100	80
		50	100	100	100	100	56	100	80
		After half samples	100	100	100	100	56	100	80
		After last sample	100	100	100	100	56	100	100
**Multi-robot cases**	**FVP** (n=3)	10	40	40	32	36	36	48	48
		**25**	**96**	**100**	**96**	**96**	44	**100**	64
		35	**100**	**100**	**100**	**100**	56	**100**	64
		45	100	100	100	100	64	96	60
		50	100	100	100	100	64	96	60
		After half sample	100	100	100	100	76	100	60
		After last sample	100	100	100	100	76	100	60
	**DVP** (n=3)	10	36	40	28	32	32	40	48
		**25**	**100**	**100**	**100**	**92**	56	**88**	56
		**35**	**100**	**100**	**100**	**96**	76	**100**	56
		45	100	100	100	96	88	100	60
		50	100	100	100	96	92	100	60
		After half samples	100	100	100	100	96	100	60
		After last sample	100	100	100	100	96	100	68

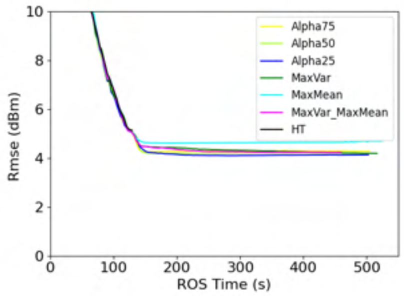

(a) RMSE

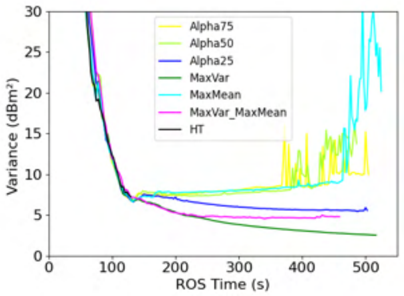

(b) Variance

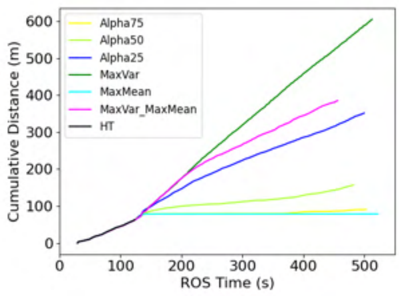

(c) Cumulative Distance

Figure S1. RMSE, Variance and Cumulative Distance for Fixed Sweep Variants for n=3.

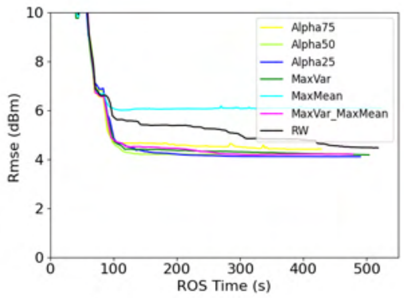

(a) RMSE

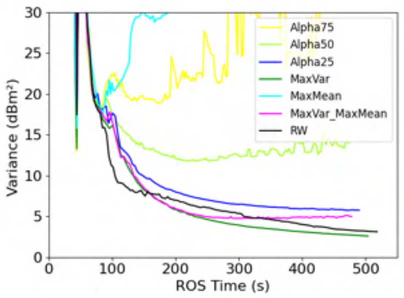

(b) Variance

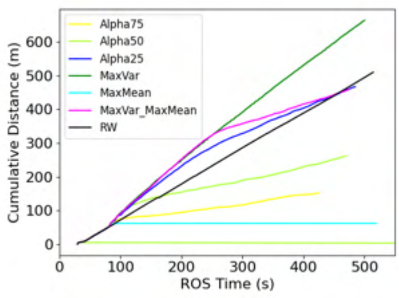

(c) Cumulative Distance

Figure S2. RMSE, Variance and Cumulative Distance for Random Walk Variants for n=3.

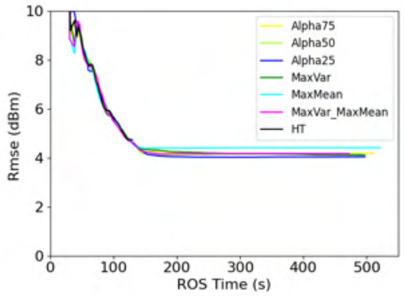

(a) RMSE

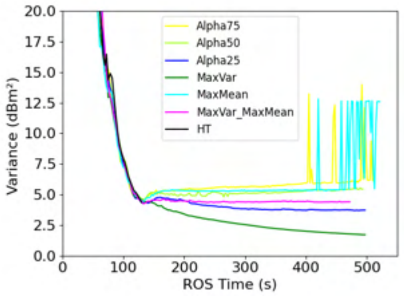

(b) Variance

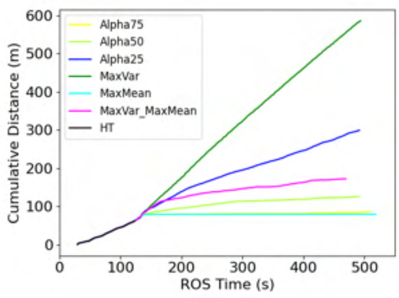

(c) Cumulative Distance

Figure S3. RMSE, Variance and Cumulative Distance for Hector Variants and n=2.

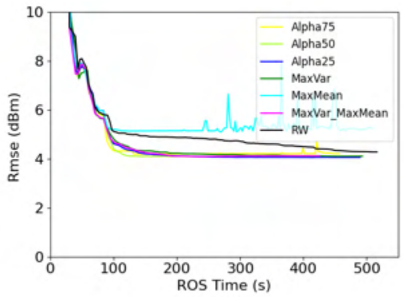

(a) RMSE

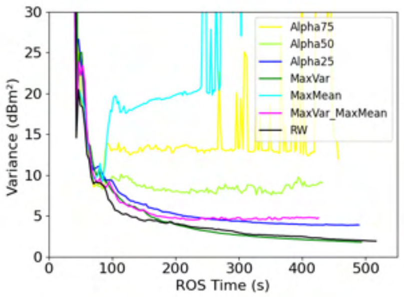

(b) Variance

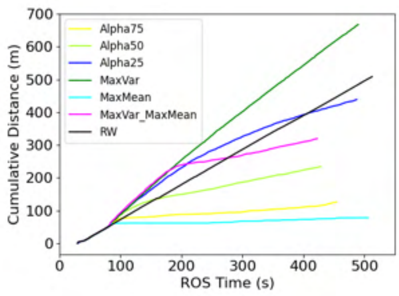

(c) Cumulative Distance

Figure S4. RMSE, Variance and Cumulative Distance for Random Walk Variants n=2.

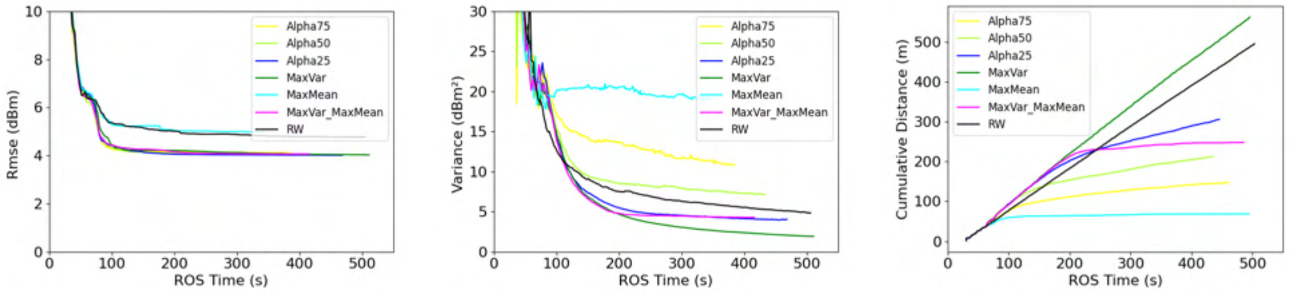

(a) RMSE (b) Variance (c) Cumulative Distance

Figure S5. RMSE, Variance and Cumulative Distance for Multi-Robots in Fixed Voronoi Partitioning.

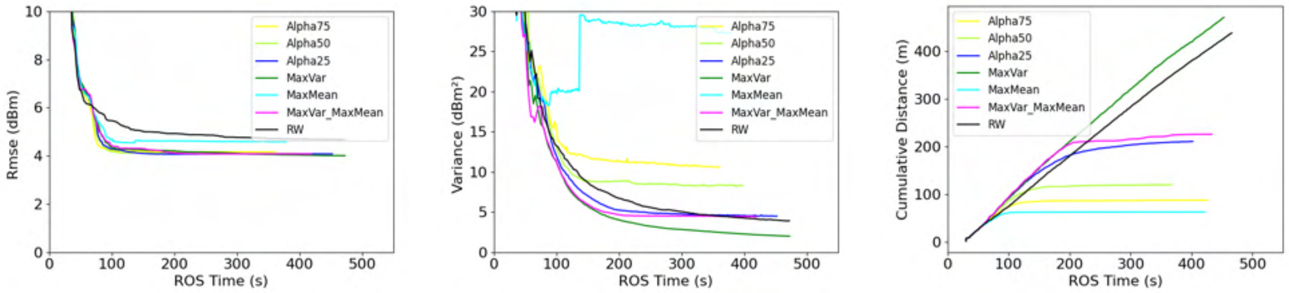

(a) RMSE (b) Variance (c) Cumulative Distance

Figure S6. RMSE, Var and Cumulative Distance for Multi-Robots in Dynamic Voronoi Partitioning.

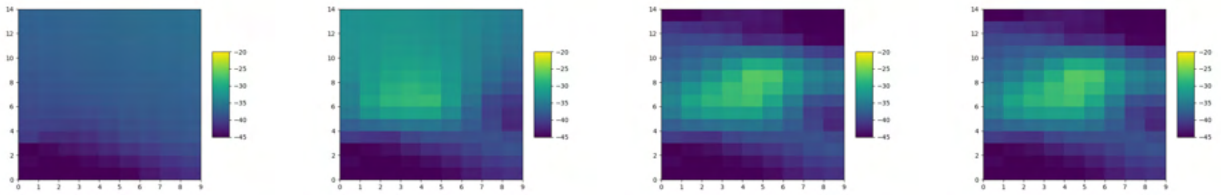

(a) After 10 Samples (b) After Fixed Sweep (c) After half exploration (d) After last sample

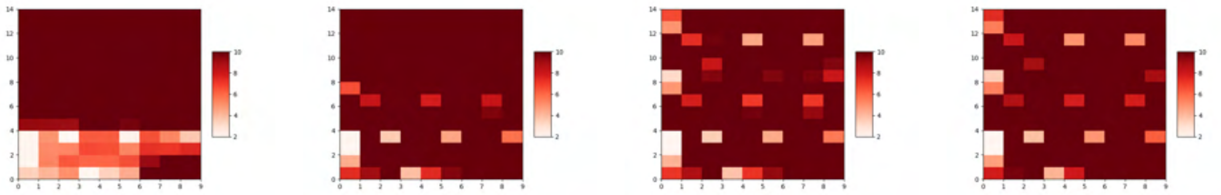

(e) After 10 Samples (f) After Fixed Sweep (g) After half exploration (h) After last sample

Figure S7. Mean and Variance for Fixed Sweep Baseline and Source location (4,7).

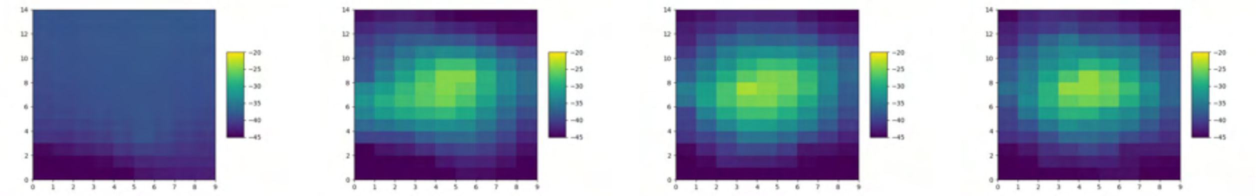

(a) After 10 Samples (b) After Fixed Sweep (c) After half exploration (d) After last sample

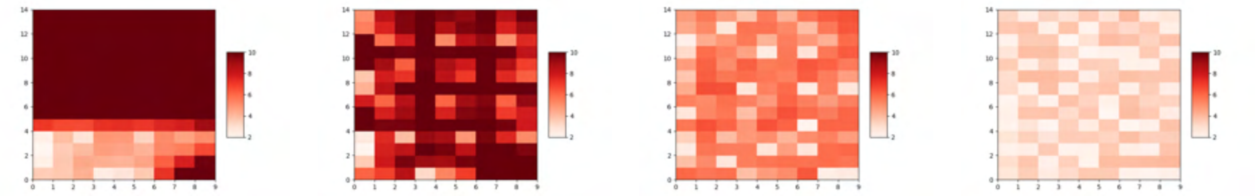

(e) After 10 Samples (f) After Fixed Sweep (g) After half exploration (h) After last sample

Figure S8. Fixed Sweep Scenario - Mean and Variance for MaxVar Approach and Source location (4,7)

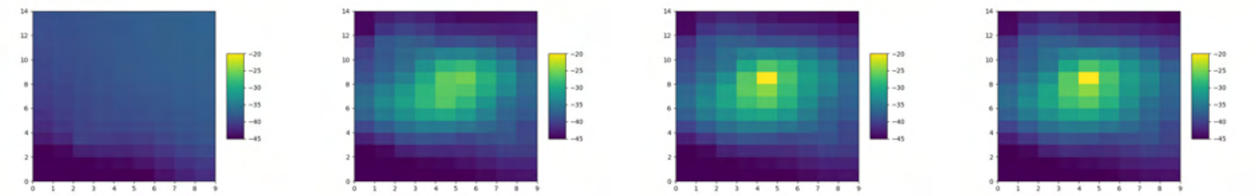

(a) After 10 Samples (b) After Fixed Sweep (c) After half exploration (d) After last sample

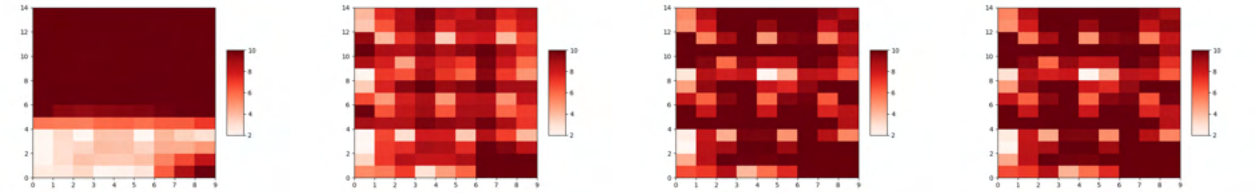

(e) After 10 Samples (f) After Fixed Sweep (g) After half exploration (h) After last sample

Figure S9. Fixed Sweep Scenario - Mean and Variance for MaxMean Approach and Source location (4,7)

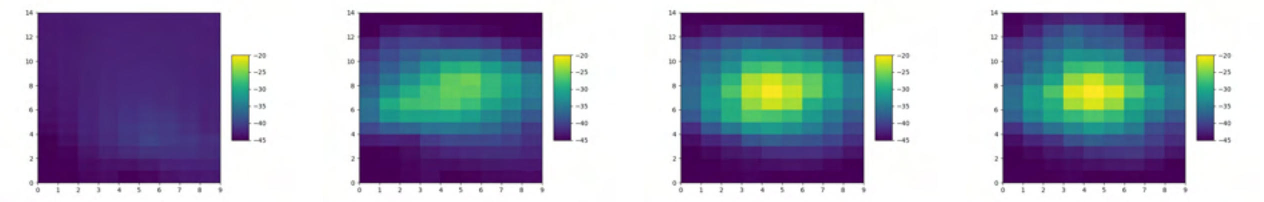

(a) After 10 Samples (b) After Fixed Sweep (c) After half exploration (d) After last sample

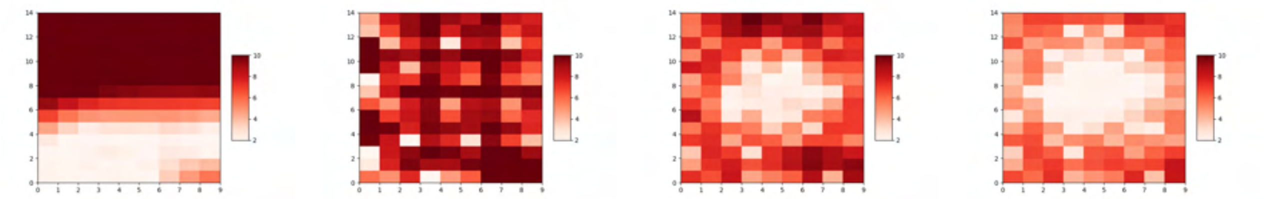

(e) After 10 Samples (f) After Fixed Sweep (g) After half exploration (h) After last sample

Figure S10. Fixed Sweep Scenario - Mean and Variance for Alpha0.25 Approach and Source location (4,7)

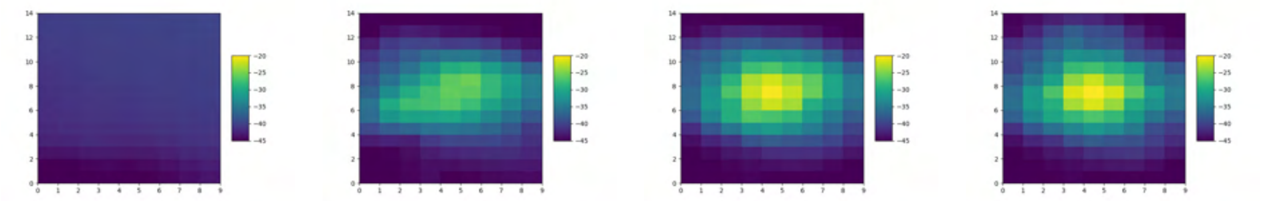

(a) After 10 Samples (b) After Fixed Sweep (c) After half exploration (d) After last sample

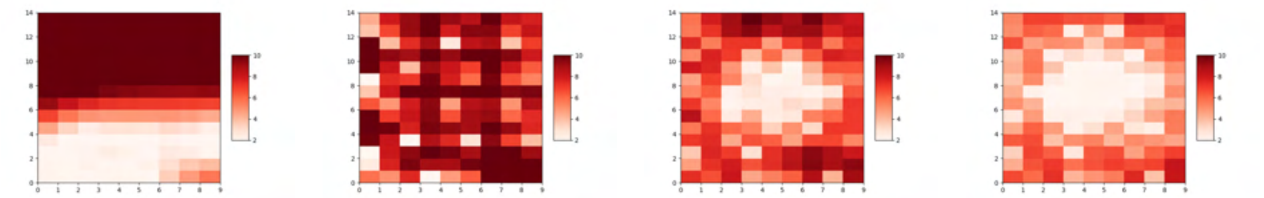

(e) After 10 Samples (f) After Fixed Sweep (g) After half exploration (h) After last sample

Figure S11. Fixed Sweep Scenario - Mean and Variance for Alpha0.5 Approach and Source location (4,7)

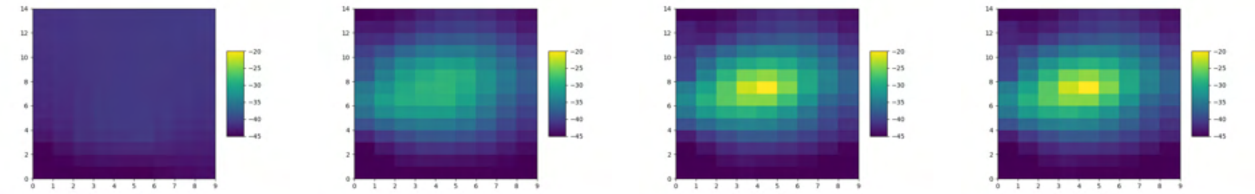

(a) After 10 Samples (b) After Fixed Sweep (c) After half exploration (d) After last sample

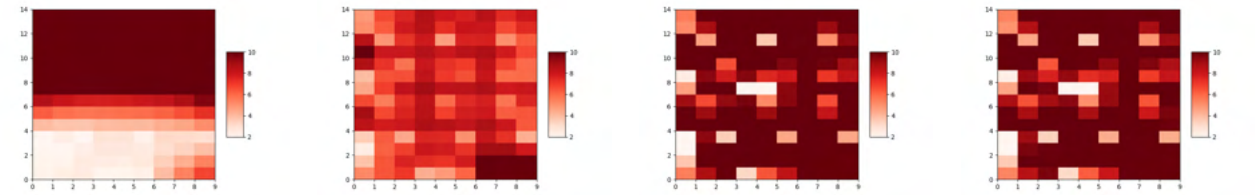

(e) After 10 Samples (f) After Fixed Sweep (g) After half exploration (h) After last sample

Figure S12. Fixed Sweep Scenario - Mean and Variance for Alpha0.75 Approach and Source location (4,7)

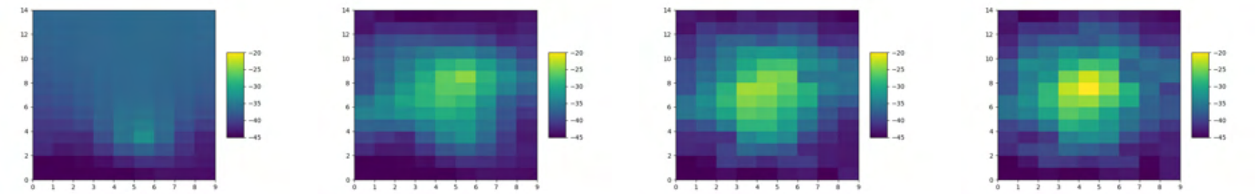

(a) After 10 Samples (b) After Fixed Sweep (c) After half exploration (d) After last sample

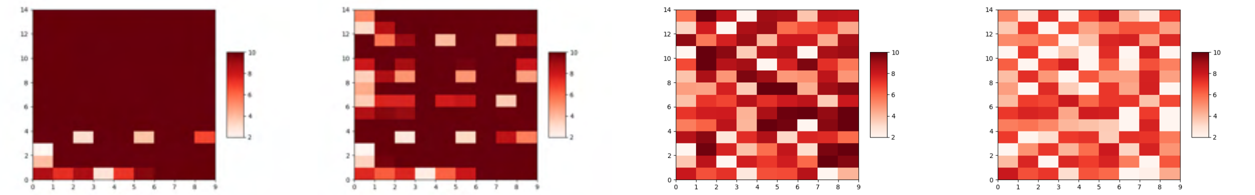

(e) After 10 Samples (f) After Fixed Sweep (g) After half exploration (h) After last sample

Figure S13. Fixed Sweep Scenario - Mean and Variance for MaxVarMaxMean Approach and Source location (4,7)

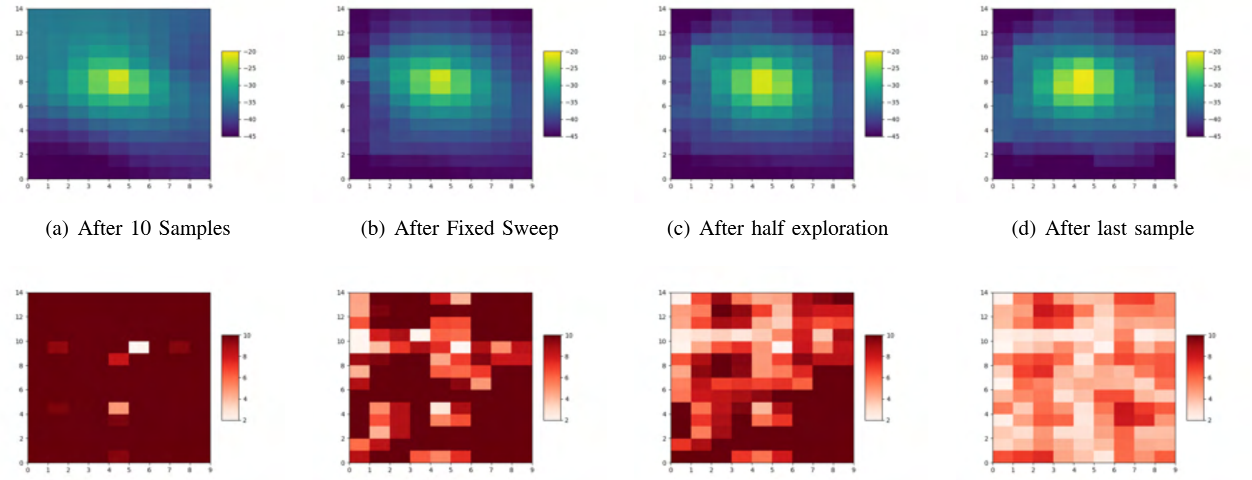

(a) After 10 Samples (b) After Fixed Sweep (c) After half exploration (d) After last sample

(e) After 10 Samples (f) After Fixed Sweep Equivalent (g) After half exploration (h) After last sample

Figure S14. Mean and Variance for Random Walk Baseline and Source location (4,7).

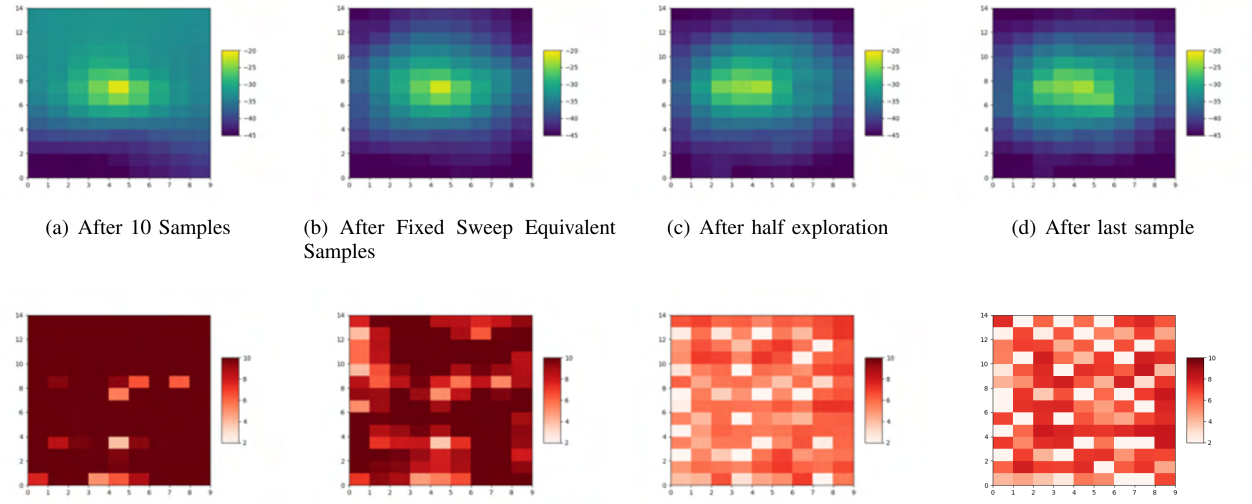

(a) After 10 Samples (b) After Fixed Sweep Equivalent Samples (c) After half exploration (d) After last sample

(e) After 10 Samples (f) After Fixed Sweep (g) After half exploration (h) After last sample

Figure S15. Random Walk Scenario - Mean and Variance for MaxVar Approach and Source location (4,7)

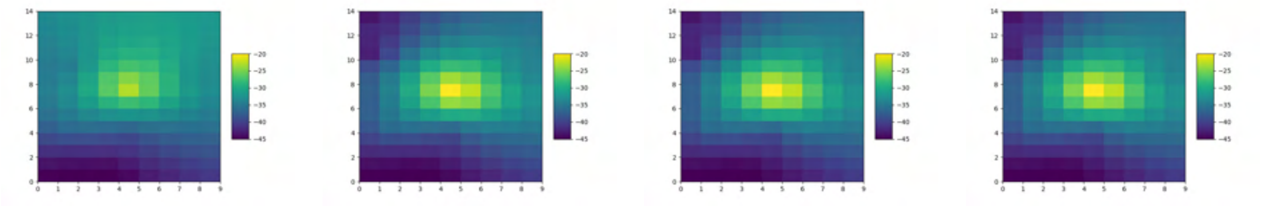

(a) After 10 Samples (b) After Fixed Sweep Equivalent Samples (c) After half exploration (d) After last sample

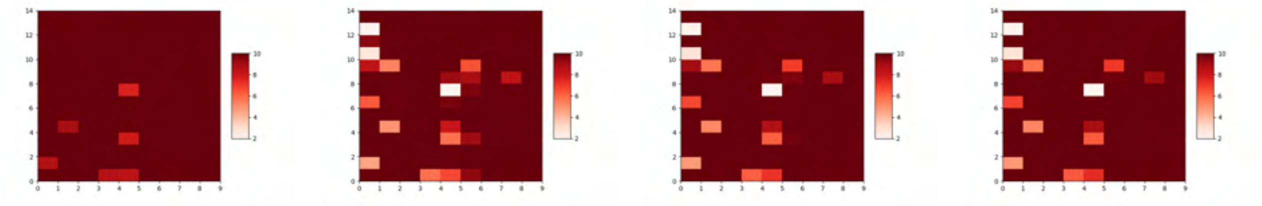

(e) After 10 Samples (f) After Fixed Sweep (g) After half exploration (h) After last sample

Figure S16. Random Walk Scenario - Mean and Variance for MaxMean Approach and Source location (4,7)

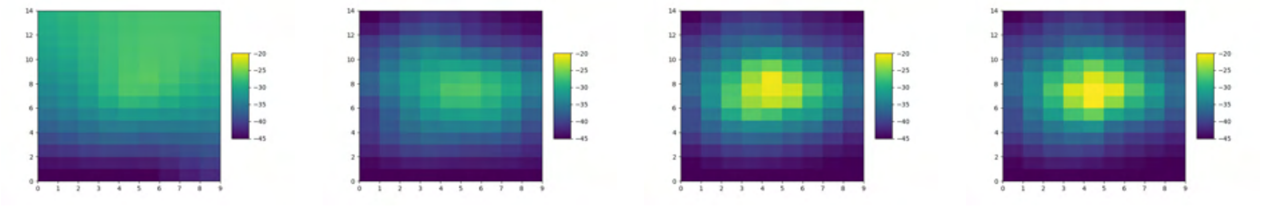

(a) After 10 Samples (b) After Fixed Sweep Equivalent Samples (c) After half exploration (d) After last sample

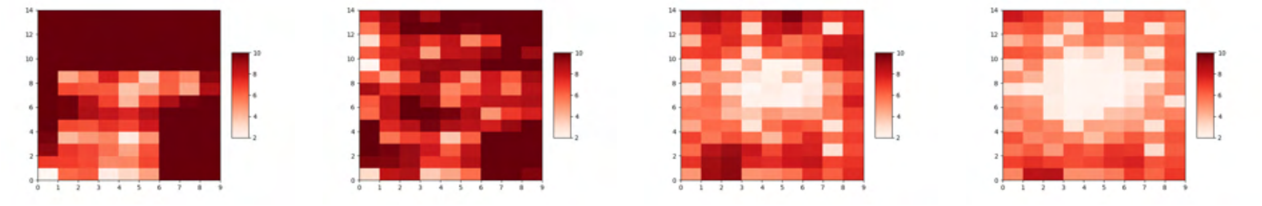

(e) After 10 Samples (f) After Fixed Sweep Equivalent Samples (g) After half exploration (h) After last sample

Figure S17. Random Walk Scenario - Mean and Variance for Alpha0.25 Approach and Source location (4,7)

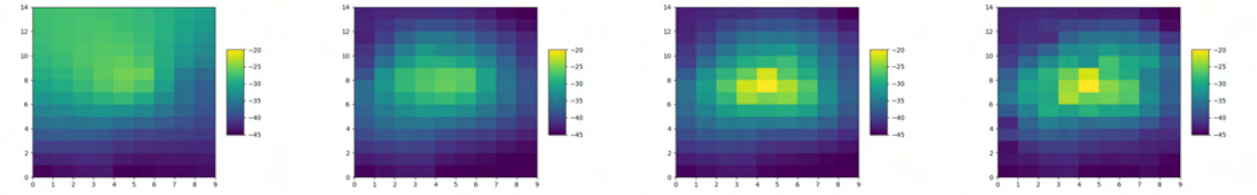

(a) After 10 Samples (b) After Fixed Sweep Equivalent Samples (c) After half exploration (d) After last sample

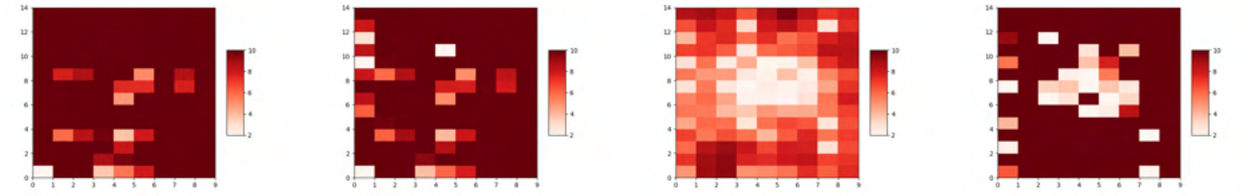

(e) After 10 Samples (f) After Fixed Sweep Equivalent Samples (g) After half exploration (h) After last sample

Figure S18. Random Walk Scenario - Mean and Variance for Alpha 0.5 Approach and Source location (4,7)

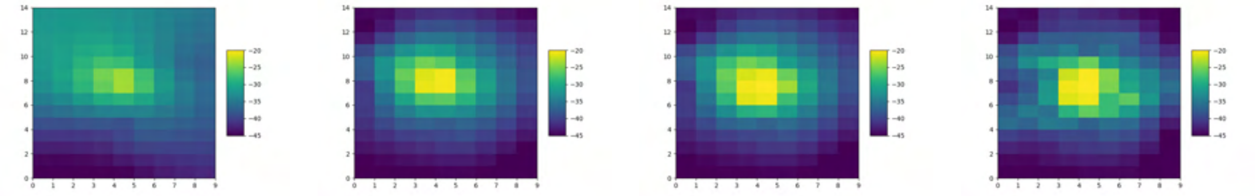

(a) After 10 Samples (b) After Fixed Sweep Equivalent Samples (c) After half exploration (d) After last sample

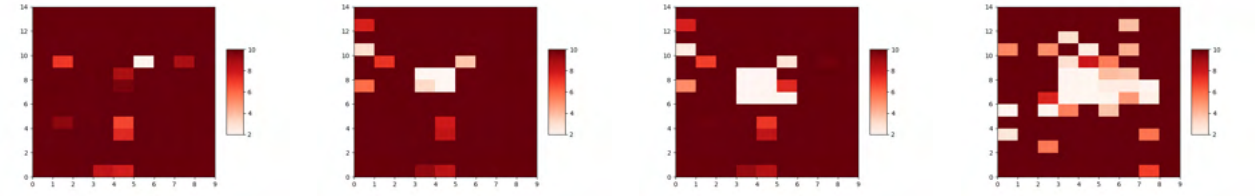

(e) After 10 Samples (f) After Fixed Sweep Equivalent Samples (g) After half exploration (h) After last sample

Figure S19. Random Walk Scenario - Mean and Variance for Alpha0.75 Approach and Source location (4,7)

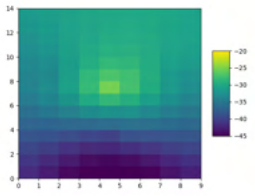

(a) After 10 Samples

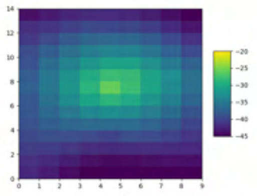

(b) After Fixed Sweep Equivalent Samples

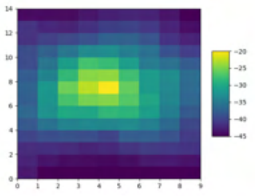

(c) After half exploration

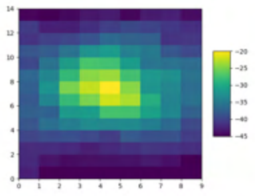

(d) After last sample

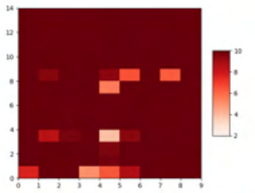

(e) After 10 Samples

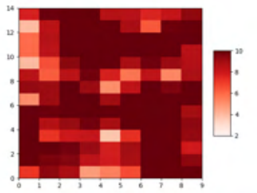

(f) After Fixed Sweep

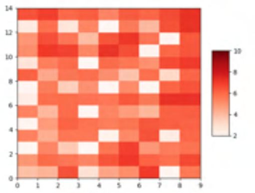

(g) After half exploration

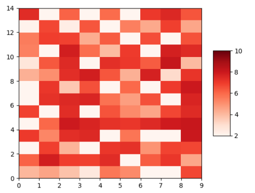

(h) After last sample

Figure S20. Random Walk Scenario - Mean and Variance for MaxVarMaxMean Approach and Source location (4,7)

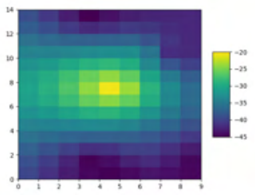

(a) After 10 Samples

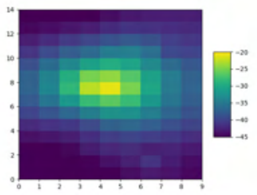

(b) After Fixed Sweep

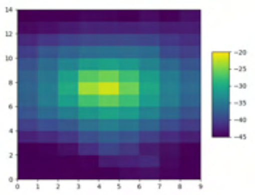

(c) After half exploration

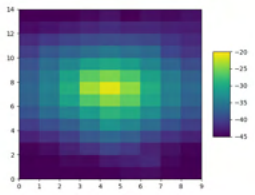

(d) After last sample

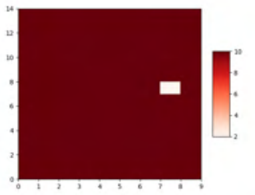

(e) After 10 Samples

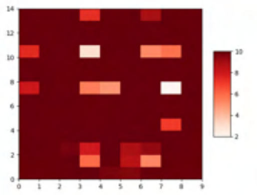

(f) After Fixed Sweep

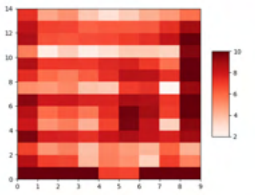

(g) After half exploration

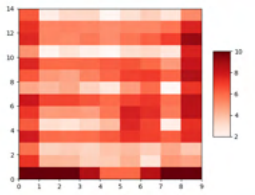

(h) After last sample

Figure S21. Fixed Voronoi Partition - Mean and Variance for Random Walk Baseline and Source location (4,7)

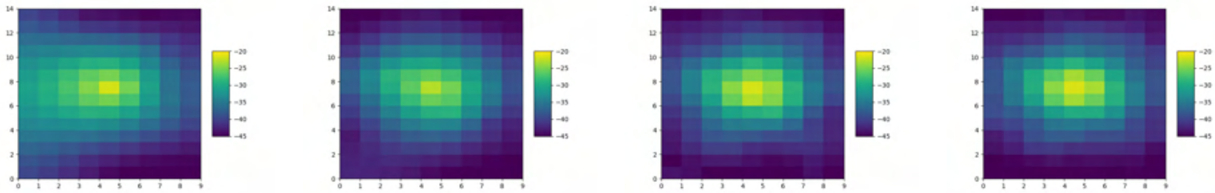

(a) After 10 Samples (b) After Fixed Sweep (c) After half exploration (d) After last sample

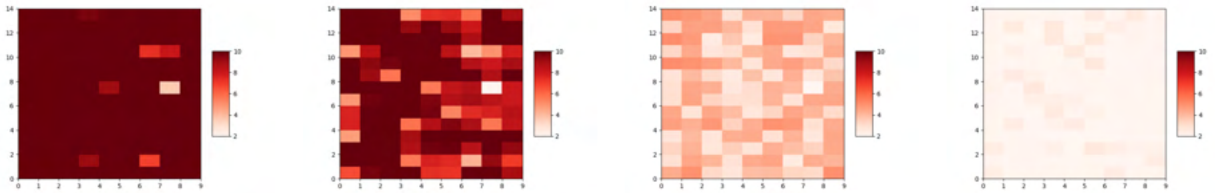

(e) After 10 Samples (f) After Fixed Sweep (g) After half exploration (h) After last sample

Figure S22. Fixed Voronoi Partition - Mean and Variance for MaxVar Approach and Source location (4,7)

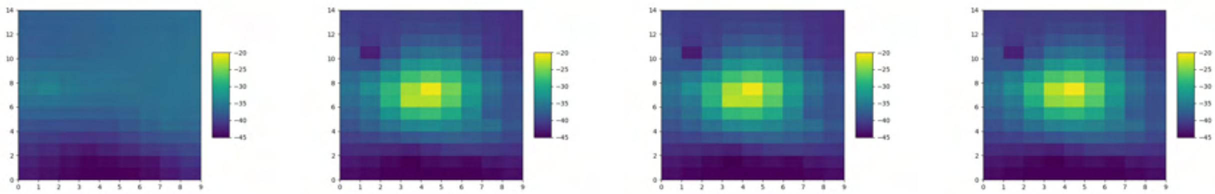

(a) After 10 Samples (b) After Fixed Sweep (c) After half exploration (d) After last sample

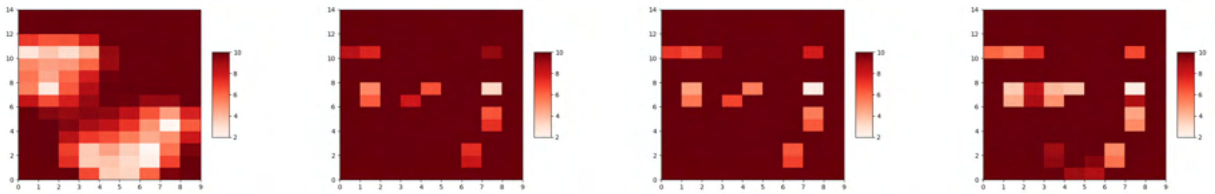

(e) After 10 Samples (f) After Fixed Sweep (g) After half exploration (h) After last sample

Figure S23. Fixed Voronoi Partition - Mean and Variance for MaxMean Approach and Source location (4,7)

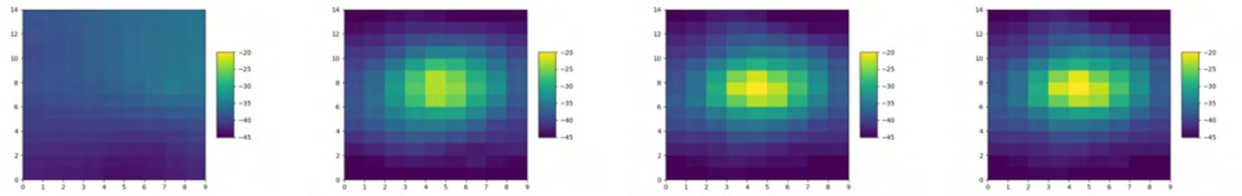

(a) After 10 Samples (b) After Fixed Sweep (c) After half exploration (d) After last sample

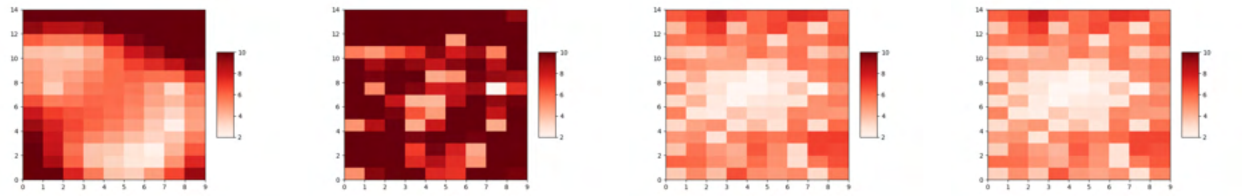

(e) After 10 Samples (f) After Fixed Sweep (g) After half exploration (h) After last sample

Figure S24. Fixed Voronoi Partition - Mean and Variance for Alpha0.25 Approach and Source location (4,7)

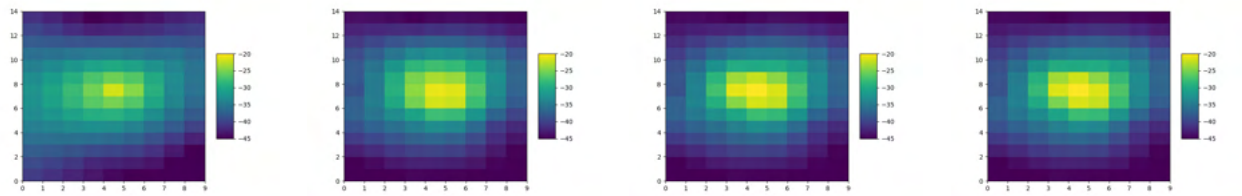

(a) After 10 Samples (b) After Fixed Sweep (c) After half exploration (d) After last sample

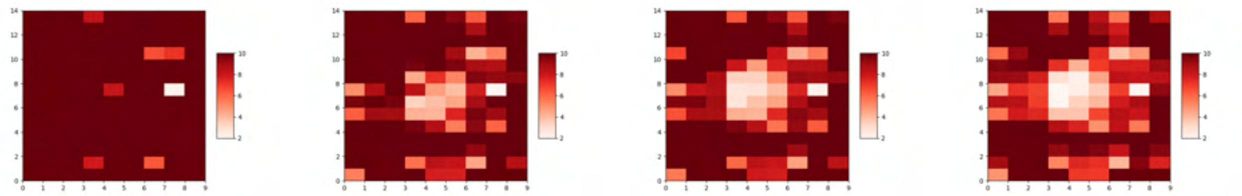

(e) After 10 Samples (f) After Fixed Sweep (g) After half exploration (h) After last sample

Figure S25. Fixed Voronoi Partition - Mean and Variance for Alpha0.5 Approach and Source location (4,7)

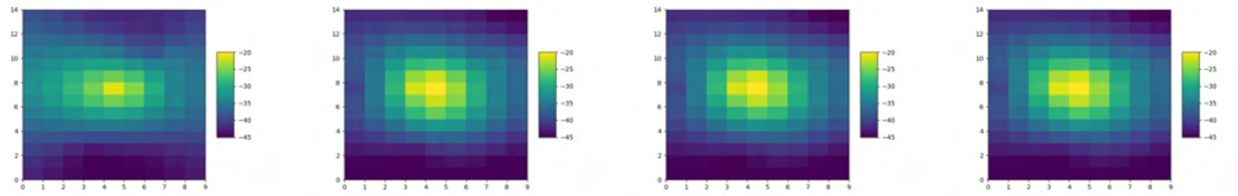

(a) After 10 Samples (b) After Fixed Sweep (c) After half exploration (d) After last sample

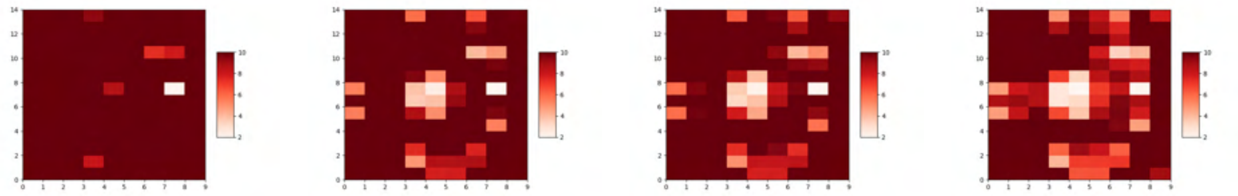

(e) After 10 Samples (f) After Fixed Sweep (g) After half exploration (h) After last sample

Figure S26. Fixed Voronoi Partition - Mean and Variance for Alpha0.75 Approach and Source location (4,7)

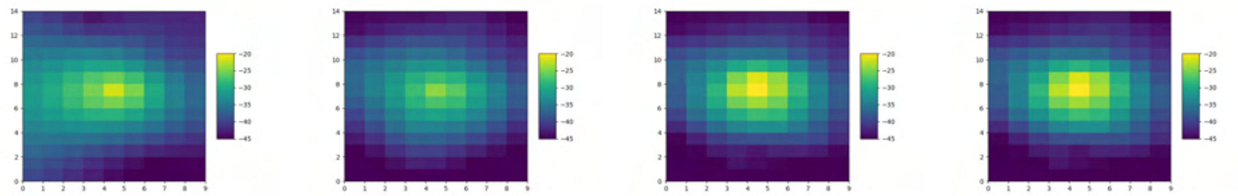

(a) After 10 Samples (b) After Fixed Sweep (c) After half exploration (d) After last sample

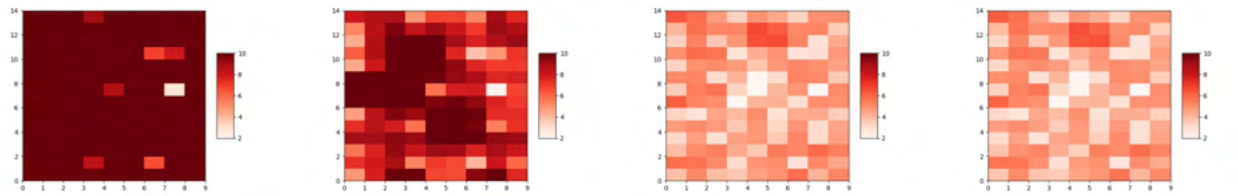

(e) After 10 Samples (f) After Fixed Sweep (g) After half exploration (h) After last sample

Figure S27. Fixed Voronoi Partition - Mean and Variance for MaxVarMaxMean Approach and Source location (4,7)

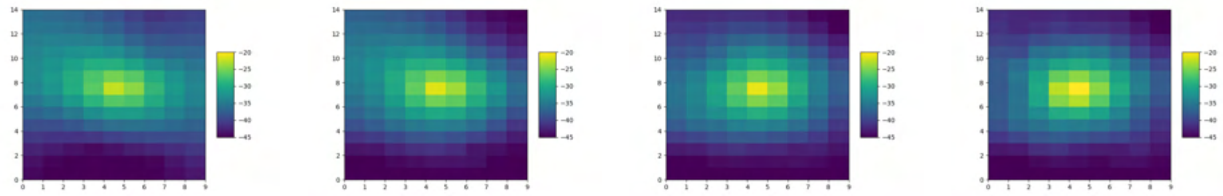

(a) After 10 Samples (b) After Fixed Sweep (c) After half exploration (d) After last sample

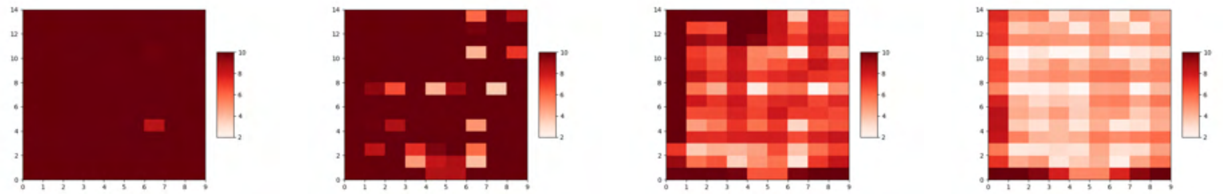

(e) After 10 Samples (f) After Fixed Sweep (g) After half exploration (h) After last sample

Figure S28. Dynamic Voronoi Partition - Mean and Variance for Random Walk Baseline and Source location (4,7)

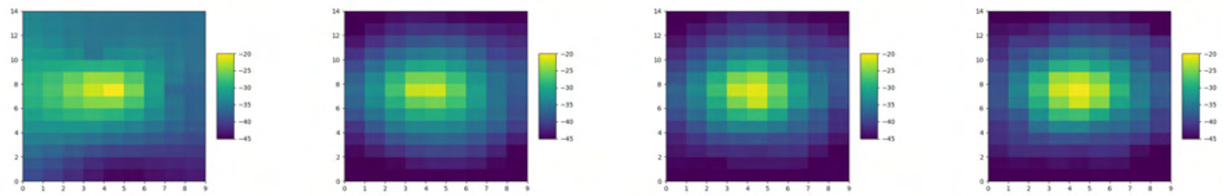

(a) After 10 Samples (b) After Fixed Sweep (c) After half exploration (d) After last sample

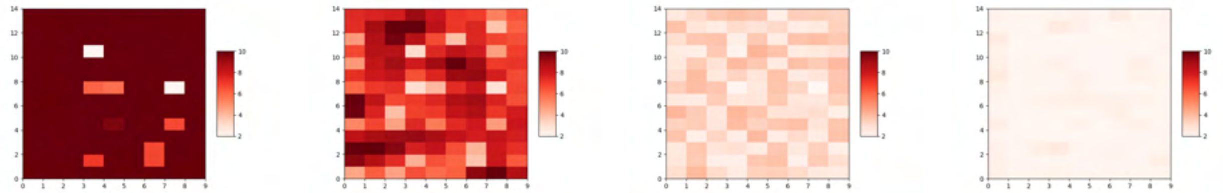

(e) After 10 Samples (f) After Fixed Sweep (g) After half exploration (h) After last sample

Figure S29. Dynamic Voronoi Partition - Mean and Variance for MaxVar Approach and Source location (4,7)

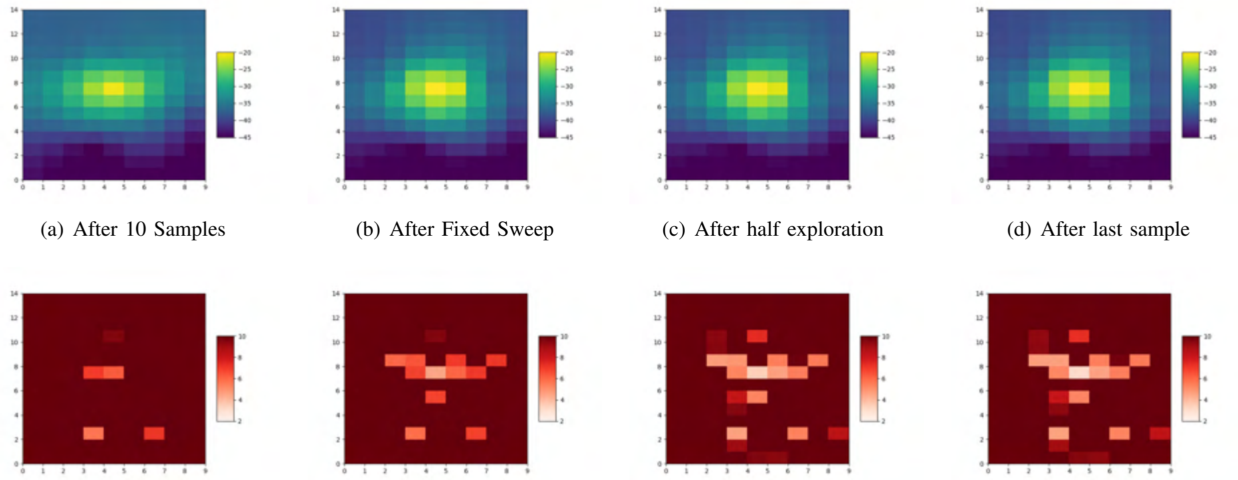

(a) After 10 Samples (b) After Fixed Sweep (c) After half exploration (d) After last sample

(e) After 10 Samples (f) After Fixed Sweep (g) After half exploration (h) After last sample

Figure S30. Dynamic Voronoi Partition - Mean and Variance for MaxMean Approach and Source location (4,7)

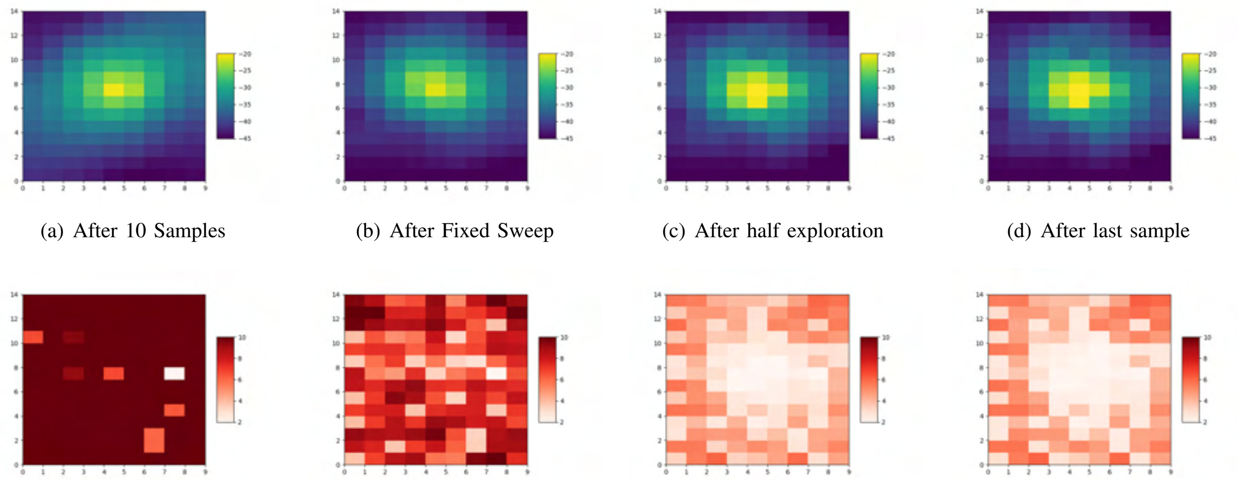

(a) After 10 Samples (b) After Fixed Sweep (c) After half exploration (d) After last sample

(e) After 10 Samples (f) After Fixed Sweep (g) After half exploration (h) After last sample

Figure S31. Dynamic Voronoi Partition - Mean and Variance for Alpha0.25 Approach and Source location (4,7)

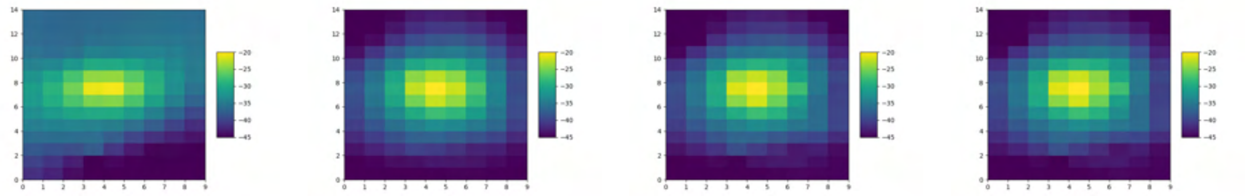

(a) After 10 Samples (b) After Fixed Sweep (c) After half exploration (d) After last sample

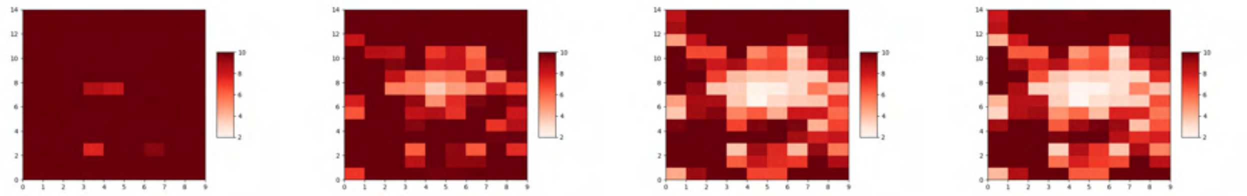

(e) After 10 Samples (f) After Fixed Sweep (g) After half exploration (h) After last sample

Figure S32. Dynamic Voronoi Partition - Mean and Variance for Alpha0.5 Approach and Source location (4,7)

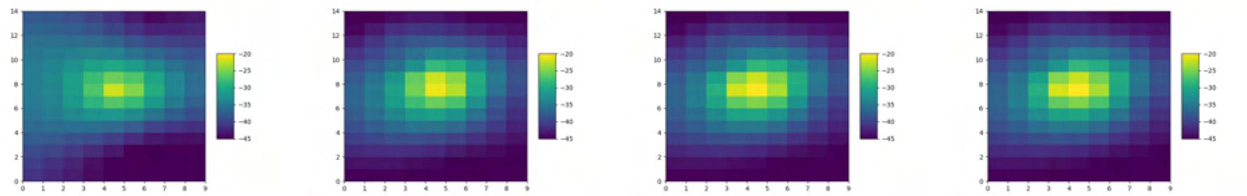

(a) After 10 Samples (b) After Fixed Sweep (c) After half exploration (d) After last sample

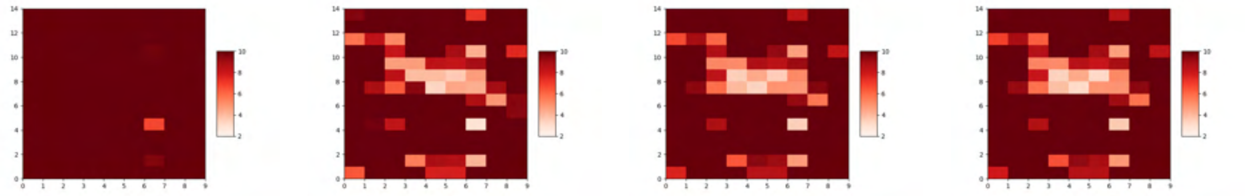

(e) After 10 Samples (f) After Fixed Sweep (g) After half exploration (h) After last sample

Figure S33. Dynamic Voronoi Partition - Mean and Variance for Alpha0.75 Approach and Source location (4,7)

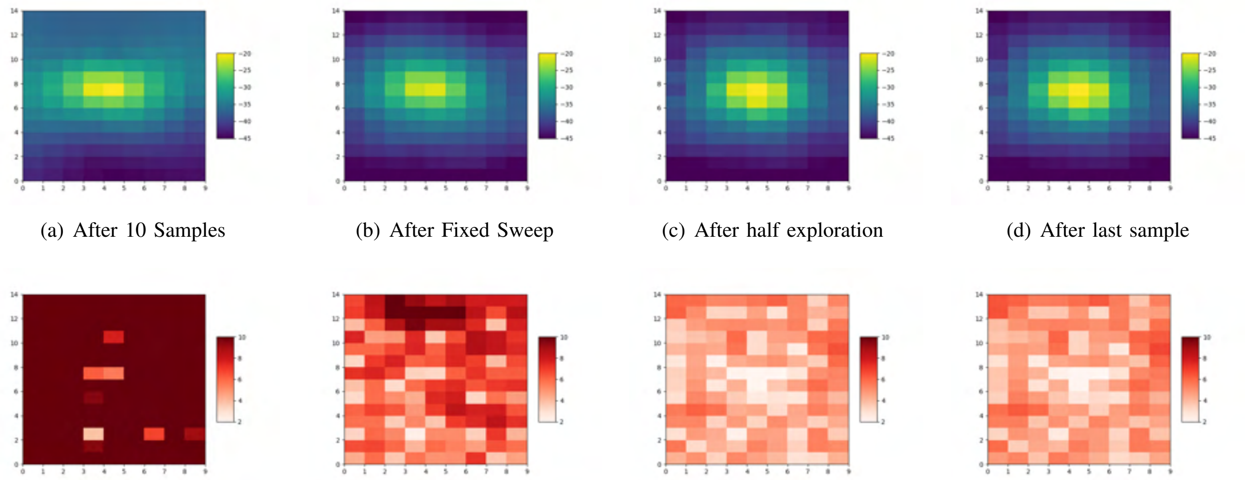

(a) After 10 Samples (b) After Fixed Sweep (c) After half exploration (d) After last sample

(e) After 10 Samples (f) After Fixed Sweep (g) After half exploration (h) After last sample

Figure S34. Dynamic Voronoi Partition - Mean and Variance for MaxVarMaxMean Approach and Source location (4,7)
